# Global distribution of cattle, horses, goats, sheep and buffaloes at 1 km resolution for 2000–2022 based on subnational census data and spatiotemporal machine learning

**DOI:** 10.7717/peerj.21494

**Published:** 2026-07-17

**Authors:** Leandro Parente, Steffen Ehrmann, Tomislav Hengl, Steffen Fritz, Carmelo Bonannella, Žiga Malek, Carlos Gonzalez Fischer, Katya Perez, Radost Stanimirova, Carsten Meyer, Dominik Wisser, Giuseppina Cinardi, Lindsey Sloat

**Affiliations:** 1 OpenGeoHub Foundation, Doorwerth, Gelderland, Netherlands; 2 German Centre for Integrative Biodiversity Research (iDiv), Leipzig, Germany; 3 Institute of Biology, Universität Leipzig, Leipzig, Germany; 4 International Institute for Applied Systems Analysis (IIASA), Vienna, Austria; 5 Biotechnical Faculty, University of Ljubljana, Ljubljana, Slovenia; 6 Department of Global Development, Cornell University, Ithaca, United States; 7 World Resources Institute, Washington, D.C., United States; 8 Animal Production and Health Division, Food and Agriculture Organization of the United Nations|FAO, Rome, Italy

**Keywords:** Census data, Livestock, Machine learning, Areal regression, Agriculture, Random forest, Gradient boosting tree, Spatiotemporal modeling, Global mapping, Open data

## Abstract

The article describes the production and evaluation of annual livestock densities and headcounts of cattle, horses, sheep, goats and buffaloes (including 95% probability prediction intervals) at 1 km spatial resolution for the 2000–2022 period using spatiotemporal machine learning. A compilation of subnational livestock census data has been imported, harmonized and used as reference data (55,336 census polygons and 939,257 individual data entries; covering 147 countries) to build predictive models. A large stack of multi-source harmonized raster data sets (128 individual layers) were used as features. Models were fitted using scikit-map and scikit-learn libraries with recursive feature elimination and Poisson criteria to represent the distribution of the target variable. Intermediate rasters estimating potential land for livestock production based on grassland and cropland extent, along with biophysical features, were used to estimate the spatial domain of livestock. The final predictions at 1 km were further adjusted to annual headcounts based on Food and Agriculture Organization Corporate Statistical (FAOSTAT) national database to ensure consistency. Model benchmarking based on 10% test samples (with spatial blocking) shows that Random Forest outperforms Gradient Boosting Tree for predicting livestock densities, with concordance correlation coefficient (CCC) values of 0.603, 0.547, 0.622, 0.598, 0.689, and Root Mean Squared Error (RMSE) values of 104.59, 6.06, 67.57, 64.09, 30.37 (heads per km^2^) for cattle, horses, sheep, goats and buffaloes. Feature importance analysis shows that the key variables include climate and socio-economic layers, such as water vapor, aridity index, land surface temperature, travel time to the nearest cities, and the spatial distribution of religious groups. Further evaluation of the output layers shows similar distributions to existing global livestock products (FAO Gridded Livestock of The World—GLW, and Annual Gridded Livestock of the World—AGLW). The spatial domain of livestock (active grazing/forage areas) is often difficult to validate, with many countries having very specific management cultures that can not be seamlessly represented using existing global raster layers, hence modeling distribution of livestock per country using local country-specific features (instead of using global models) could help increase accuracy, specially for regional/local applications. The modeling pipeline is open source and available on GitHub (https://github.com/wri/global-pasture-watch) with output layers (both original ML predictions and FAOSTAT-adjusted values) publicly available under Creative Commons Attribution (CC-BY) license on Zenodo (https://doi.org/10.5281/zenodo.17491242).

## Introduction

Livestock systems constitute a critical interface between human societies and natural environments (Haberl et al., 2019). Livestock production is a cornerstone of global food systems, providing essential animal protein while supporting the livelihoods, social cohesion, and economic stability of more than a billion people around the world (Robinson et al., 2011; Herrero et al., 2013; FAO, 2018). Livestock production is also deeply intertwined with land systems, influencing soil fertility through the input of manure and shaping vegetation dynamics (Thornton, 2010). However, livestock production also has significant environmental impacts, including land-use change, loss of biodiversity, zoonotic disease, and greenhouse gas (GHG) emissions. Domesticated livestock collectively outweigh all wild terrestrial and marine mammals by a factor of 20–40, with cattle alone contributing 400 million tons of biomass (Greenspoon et al., 2023; FAO, 2022). Additionally, methane (CH_4_) emissions from ruminant digestion are a major contributor to anthropogenic GHG emissions and are projected to further constrain global carbon budgets (MacLeod et al., 2018; Reisinger et al., 2021).

The increasing global demand for animal-based products, driven by population growth, urbanization, and rising incomes, has led to a shift towards intensified meat and dairy production systems (Alexandratos & Bruinsma, 2012; Mehrabi et al., 2020). This trend often favors large-scale industrial operations over small-scale production, exacerbating the marginalization of traditional smallholder farmers (Mgbenka, Mbah & Ezeano, 2016; Herrero et al., 2023). Furthermore, expansion of grazing land to accommodate growing demand remains a key driver of deforestation and conversion, particularly in tropical regions, with profound consequences for carbon sequestration and habitat loss (Gibbs et al., 2010; de Area Leão Pereira et al., 2020). However, well-managed pastures, often combined with agroforestry solutions, have been shown to enhance soil carbon sequestration (de Freitas et al., 2020). Livestock production systems vary worldwide, creating the need for targeted and regionally relevant measures to improve smallholder incomes, reduce environmental impacts, and ensure food security (Farooq et al., 2022). Accordingly, detailed spatially explicit data sets on livestock are crucial for addressing complex socioeconomic and environmental challenges (Kazanski et al., 2025). However, assessing the environmental and socioeconomic impacts of livestock has been challenging due to the lack of sufficiently high resolution, frequently updated, and globally consistent livestock distribution data.

The main source of spatial data for global livestock is the Gridded Livestock of the World (GLW) (Wisser & Cinardi, 2024), which FAO and several other organizations officially adopt. The latest version, GLW v4, has a spatial resolution of 10 km and is centered around 2020. The data set is based on a Random Forest framework developed by Nicolas et al. (2016) and Gilbert et al. (2018), which downscales livestock census data to 1 km resolution, although only coarser 10 km resolution raster layers are currently publicly available. More recently, the annual gridded livestock of the world (AGLW) has become an important resource for global livestock, providing annual densities (*i.e*., heads per km^2^) from 1961 to 2021 at 5 km spatial resolution for cattle, buffaloes, sheep, goats, horses, pigs, chickens, and ducks with uncertainty quantification and fully compliant with FAOSTAT nationally reported statistics (Du et al., 2025). Other studies have further refined regional livestock mapping: Meisner et al. (2022) improved the downscaling of livestock counts from census data for Malawi, Uganda, the Democratic Republic of Congo (DRC) and South Sudan; Donovan et al. (2024) produced high-resolution livestock densities for sheep, beef and dairy cattle distribution in New Zealand for the year 2017; Kolluru et al. (2023) developed a gridded data set of horses, sheep and goats for Kazakhstan at 1 km spatial resolution from 2000 to 2019 using vegetation proxies and other raster layers through Random Forest regression modeling; Zhan et al. (2024) modeled annual distributions of cattle and sheep in China from 2000 to 2022 at 1 km spatial resolution, considering grazing livestock production systems, national census data and Random Forest models. All these studies show that modeling approaches combining census polygons with 1 km raster layers can substantially refine the spatial and temporal resolution of livestock distribution data sets, improving their applicability for further studies. Global remote sensing products for land cover (Potapov et al., 2022a), croplands (Potapov et al., 2022b), grasslands (Parente et al., 2024b), above-ground biomass (Araza et al., 2023), and canopy height (Lang et al., 2023; Potapov et al., 2022a) provide 30 m resolution data for almost every year since 2000, while livestock distribution raster layers are based on coarse census polygons (mean polygon size of 2,907 km^2^; SD = 21,463), so the derived rasters remain limited in spatial resolution (*i.e*., 5–10 km).

In this article, we present two data sets that are publicly available at 1 km spatial resolution from 2000 to 2022: (i) global annual livestock densities for cattle, goats, sheep, horses, and buffaloes with prediction intervals (lower and upper boundaries around mean predictions), and (ii) global annual livestock headcounts, adjusted according to FAOSTAT reported national statistics. These data sets were produced using a spatiotemporal modeling framework that leverages the largest known compilation of subnational livestock census data, a set of multi-source raster layers harmonized at 1 km resolution, and optimized machine learning models. To support the the spatial allocation of livestock, we developed intermediate raster layers of the *potential land for livestock* from estimates of grassland (Parente et al., 2024b) and cropland extent (Potapov et al., 2022b), along with a range of carefully selected terrain, climate, land cover, socio-economic and remote sensing variables. These data sets, produced as part of the Land & Carbon Lab project (https://landcarbonlab.org), are intended to be regularly updated alongside improvements to global grassland products and are publicly available as open data under the CC-BY license from https://doi.org/10.5281/zenodo.14933636; the open source code is available *via* https://github.com/wri/global-pasture-watch. Portions of this text were previously published as part of a preprint (https://doi.org/10.21203/rs.3.rs-6201916/v1).

## Materials and Methods

### Theoretical background

Modeling based on census data and raster layers of environmental and socioeconomic conditions falls within the domain of *areal regression modeling*, where the target variable is predicted per polygon or per raster cell as a continuous variable (Stevens et al., 2015; Moraga, 2019). Since census data, particularly count-based variables, often follow a Poisson distribution, predictive methods must accommodate skewed distributions. Common approaches include machine learning (ML) algorithms such as Quantile Regression Forest (Mathlouthi, Fredette & Larocque, 2015), Gradient Boosting Trees (So, 2024), Poisson Deep Neural Network (Montesinos-Lopez et al., 2021) and Integrated Nested Laplace Approximation (INLA) (Moraga, 2019). Alternatively, the target variable can be transformed into an asymptotically normal distribution, enabling the use of Generalized Linear Models (GLMs), Geographically Weighted Regression, and similar methods suited for normally distributed data (Walker, 2023).

Two main ML-based modeling approaches are commonly used to integrate census-based response variables (available as polygons) with raster data used as predictive variables (Metzger et al., 2022; Walker, 2023):

1. **Areal regression**: the raster layers are aggregated in each census polygon with a mass-preserving statistic (typically the mean); an ML model is trained on those polygon-level records and then applied to every raster cell to downscale the predictions (Stevens et al., 2015; Li, Hou & Huang, 2021; Metzger et al., 2022). The approach assumes that aggregation truly preserves mass, and its accuracy falls off when polygons are very large or heterogeneous, because spatial variation is smoothed and the size of the polygon is often ignored (Nicolas et al., 2016; Gábor et al., 2020).
2. **Simulation-based point sampling**: within each polygon, a probability sample (*e.g*., simple-random, systematic, stratified) of points is drawn; every point inherits the polygon’s response value, and raster layers are extracted at their native raster cell resolution. Then an ML model is trained on this synthetic point set and applied to each cell of the target raster output (Gábor et al., 2020; Moraga, 2019). Because all points in the polygon share the same response, the data are pseudo-replicated; large polygons dominate the loss function, the effective sample size is overstated, and cross-validation at the point-level inflates accuracy (Gábor et al., 2020). Predictions are not forced to reconcile with the original area unit totals, and an unbalanced sampling scheme may miss environmentally distinct pockets inside big polygons, so weighting, polygon-blocked validation, and mass-preservation post-processing are advisable.

### Modeling framework overview

In this study, we implemented an areal regression modeling framework to estimate global livestock densities using subnational census data and spatiotemporal ML. We harmonized—to our knowledge—the largest global collection of livestock census data comprising 147 countries and 55,336 administrative units and covering (sparsely) 23 years (*i.e*., 2000–2022). The global densities of cattle, goats, sheep, horses, and buffaloes were modeled by combining the census data with 128 environmental, socioeconomic, and anthropogenic raster layers provided at different temporal resolutions *e.g*., monthly, annual, long-term, and static variables. To establish the modeling spatial domain, we integrated Landsat-based products of cultivated and natural/semi-natural grasslands (Parente et al., 2024b) and cropland extent (Potapov et al., 2022b), restricted by limiting land surface temperature, into a time series of potential land for livestock production (2000–2022) at 1 km spatial resolution. The final ML models, selected after a comprehensive model benchmarking, were used to produce annual livestock densities, including prediction intervals per raster cell (lower and upper boundaries around mean predictions). Lastly, the predicted densities were used to derive global headcounts at the same spatial and temporal resolution, matching with country-level livestock counts provided by FAOSTAT. Harmonization of census data was implemented in the R programming language while the modeling framework was implemented in Python; the entire workflow was run on a single CPU server with 1 TB of RAM and 96 processing threads.

**Figure 1:**
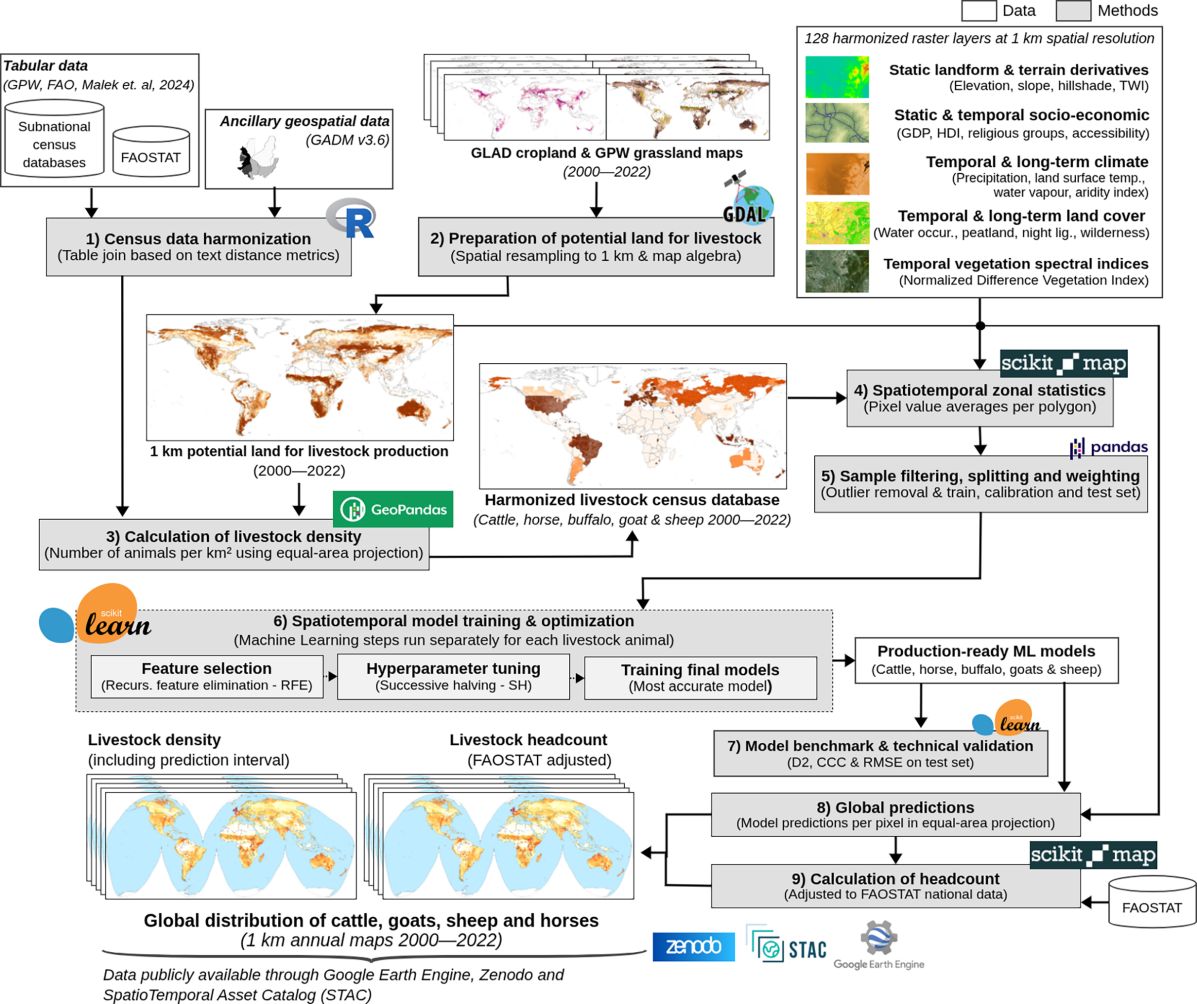
Spatiotemporal modeling framework implemented for producing the global annual distribution of cattle, goats, sheep, horses and buffaloes, including prediction intervals (lower and upper boundaries around mean predictions), for 2000–2022 at 1 km spatial resolution. Corresponding Python and R libraries are indicated using logos. See text for more details.

The primary modeling steps are illustrated in Fig. 1 and outlined below; subsequent subsections provide a detailed description of the entire methodology.

1. **Harmonization of census data**: Census data from various national agencies were downloaded, curated and standardized using an ontology framework (Table S1), and later integrated with existing polygonal data of livestock (Malek et al., 2024; Wisser & Cinardi, 2024). The tabular data were then joined with the boundaries of the administrative area polygons (at national and subnational levels, depending on availability), with each polygon having assigned headcounts for each livestock species and reference year.
2. **Preparation of potential land for livestock**: We conducted the integration of cultivated and natural/semi-natural grasslands (Parente et al., 2024b) and cropland extent (Potapov et al., 2022b) considering the fractions of land use within the 1 km^2^ raster cell. For this, a global data set of agricultural production systems (Venier-Cambron et al., 2024) was used to establish fraction thresholds for livestock supported by: (i) natural/semi-natural grassland systems, (ii) cultivated grassland systems, (iii) mosaic cropland and grassland systems, and (iv) cropland systems. The annual time series (2000–2022) of the integrated potential fraction of land used for livestock production rasters at 1 km spatial resolution contained values in the range 1–100%.
3. **Calculation of livestock density**: For each census polygon, the reference year was used to match the annual layer of potential land for livestock production and to estimate the area under livestock production, adding up all raster cell fractions inside the polygon boundaries. The estimated area was used together with the headcount to estimate yearly livestock densities for each species in all polygons.
4. **Spatiotemporal zonal statistics**: For each census polygon with livestock density values, mean estimates for all harmonized raster layers were computed using only the cells inside the potential land for livestock production and polygon boundaries. For annual variables (*e.g*., Normalized Difference Vegetation Index, precipitation, Human Development Index), the reference year was used to match the raster layers, producing a sample data set composed of annual livestock density values and spatiotemporal mean features.
5. **Sample filtering, splitting, and weighting**: An outlier removal approach was implemented (see Table 1) to filter the highest quality census polygons. The data sets were then randomly split into training (80%), calibration (10%), and testing (10%) data sets, considering a blocking strategy based on regular tile systems (~200 × 200 km), keeping all polygons intersecting a specific tile only in one split set. A sample weighting scheme was applied to account for polygon size variations and was evaluated in the model benchmarking.
6. **Model training and optimization**: Two ML models were trained and optimized: Quantile Regression Forest (QRF) (Mathlouthi, Fredette & Larocque, 2015) and Gradient Boosting Trees (GBT) (So, 2024); both considering 128 predictor variables extracted *via* spatiotemporal zonal statistics. Optimization was based on the calibration set polygons and included: (i) selection of the most important features using Recursive Feature Elimination (RFE) (Demarchi et al., 2020), and (ii) hyper-parameter search/tuning using Successive Halving (SH) (Jamieson & Talwalkar, 2016). The best features and hyper-parameters were then used to train the final model using 90% of census polygons (train & calibration sets combined).
7. **Model benchmarking and technical validation**: Testing set polygons were used to estimate the D^2^ regression score (O’Brien, 2016) (suitable for skewed distributions), root mean square error (RMSE) and Concordance Correlation Coefficient (CCC) (Steichen & Cox, 2002) for each trained model. All models were benchmarked and the most accurate was selected for running the global predictions for each livestock species.
8. **Global predictions**: Annual densities for cattle, goats, sheep, horses, and buffaloes were predicted at 1 km spatial resolution using the final ML models, accompanied by lower and upper prediction limits derived from QRF (*i.e*., 2.5th and 97.5th percentiles—95% probability distribution). The predicted densities were multiplied by the fraction of land suitable for livestock production to derive raw predictions of global headcounts.
9. **Calculation of headcount**: Using a country-by-country scaling approach (see Eq. (6)), the global headcount raster cell values were adjusted. This scaling process ensured that the aggregated total of the new values matched the national statistics of FAOSTAT (version 2024.06).

**Table 1:** Number of samples (*e.g*., spatiotemporal census polygons) used for modeling livestock densities. Percentiles were estimated per species and using all census polygons.

Livestock species	Outlier thres. (98th perc.)	Outliers samples	Zero imputed (2nd perc.)	Total
Cattle	1,511 heads/km^2^	6,247	7,398	313,604
Horses	83 heads/km^2^	3,331	7,401	170,628
Goats	832 heads/km^2^	3,211	15,154	172,536
Sheep	713 heads/km^2^	3,883	8,193	198,465
Buffaloes	490 heads/km^2^	1,090	30,636	84,024

### Preparation of harmonized raster layers

The livestock modeling framework integrated static and temporal variables as input features, with static variables representing long-term (*e.g*., 20-year average land surface temperature) and mostly invariant characteristics of the landscape for the analysis period (*e.g*., elevation, slope, accessibility metrics) and temporal variables representing dynamic factors that influence livestock distributions over time (*e.g*., monthly vegetation index, annual HDI). This combination enabled the identification of structural and transient drivers of livestock densities across different regions and periods, resulting in a total of 128 individual raster layers harmonized at 1 km spatial resolution. The layers were organized into five thematic groups:

- **Static landform and terrain derivatives**: The elevation of the terrain was characterized using the global ensemble digital terrain model product (GEDTM30) provided at a resolution of 960 m (Ho et al., 2025), which integrates various global and national DTM data sets. Furthermore, we incorporated first- and second-order terrain derivatives, such as slope (Ho & Hengl, 2025b), hillshade (Ho & Hengl, 2025a) and topographic wetness index (Ho & Hengl, 2025c).
- **Static and temporal socio-economic**: To characterize human activities, which significantly influence livestock distribution by shaping land use, market access, and infrastructure development, we integrated several socioeconomic indicators in the modeling (*e.g*., population density, Gross Domestic Product (GDP) *per capita*, accessibility to cities and ports, human footprint indices, religious population). Gridded GDP *per capita* data from 1990 to 2022 were sourced from Kummu, Kosonen & Masoumzadeh Sayyar (2025), who used subnational training data from 89 countries downscaled to a 1 km resolution to predict global values. The Human Development Index (HDI) was included using the gridded data set developed by Kummu, Taka & Guillaume (2018), which provides annual estimates from 1990 to 2015. Furthermore, urbanization levels were included *via* Harmonized Global Night Time Lights (NTL), which considered calibrated stable Air Force Defense Meteorological Satellite Program (DMSP) NTL observations from 1992 to 2013, and simulated DMSP-like from the VIIRS radiance data from 2014 onward at 1 km resolution (Li et al., 2020b). The main authors recently updated the data set to cover the period 1992–2020 on a global scale (Li et al., 2020a). To characterize the religious influence on livestock practices, we spatialized the World Religion Data (Correlates of War, 2025) on a national scale and converted them to raster format, integrating into the modeling percentages of various religious populations (*i.e*., Christianity, Judaism, Islam, Buddhism, and other religions). To assess the isolation of different regions and its correlation with livestock production systems, we used a suite of 10 global accessibility indicators computed at a 1 km resolution (Nelson et al., 2019), where class 1 represents highly accessible areas and class 9 denotes remote regions with minimal infrastructure connectivity. In addition to direct human pressure indicators, we also incorporated a wilderness layer, representing areas with low or near-zero Human Footprint values (Mu et al., 2022). This data set tracks annual changes in the extent of wilderness from 2000 to 2018, highlighting regions where natural landscapes remain largely undisturbed by human activity. The inclusion of this data set allowed us to assess the degree of landscape integrity and its relationship with livestock density, particularly in extensive pastoral systems where low human influence may play a key role.
- **Long-term and temporal climate/annual time-series**: The characterization of land surface temperature dynamics, an environmental constraint for livestock activity and forage productivity, was based on the MODIS MOD11A2 product (Wan, Hook & Hulley, 2021). We produced monthly and long-term aggregates from 2000 to 2022 for daytime and nighttime surface temperatures using the 50th percentile (median). To capture seasonal precipitation and water availability, particularly relevant in grazing regions where forage availability is mainly based on rainfall, we used NASA’s Integrated Multi-satellite Retrievals for GPM (IMERG) product, specifically the monthly liquid precipitation rate at 10 km resolution. IMERG has high accuracy, especially in monthly time steps, and is in the operation phase (Pradhan et al., 2022). In addition, we used the MODIS MCD19A2 (atmospheric) product to represent the water vapor layer, which estimates column water vapor above the ground using near-IR bands. We produced long-term aggregates from 2000 to 2022 using monthly mean composites of non-cloudy observations aggregated from daily estimates. This data set provides an important measure of atmospheric moisture, which affects both evapotranspiration and vegetation growth. For further details on the preparation of these layers, refer to Parente, Simoes & Hengl (2023) and Consoli et al. (2024). We also included a monthly long-term aridity index (*i.e*., ratio of precipitation to potential evapotranspiration) at 1 km spatial resolution, based on the FAO Penman-Monteith reference evapotranspiration equation and the WorldClim data set version 2.0 (Zomer, Xu & Trabucco, 2022). This index provides a measure of moisture availability for potential growth of a reference grassland or other specific vegetation types, and constitutes an important baseline for measuring and anticipating climatic change impacts in livestock hotspots. Following the methodology of Kilibarda et al. (2014), we incorporated *geometric temperature transformations*, derived as functions of latitude, day of the year, and elevation. These transformations produced estimates of geometric minimum and maximum temperatures per month, resulting in 24 additional variables. By integrating Earth’s geometric and temporal dynamics, these variables allow the ML model to distinguish between locations that, despite having similar temperature profiles, differ in latitudinal position or seasonal timing, capturing regional temperature variations that influence livestock management practices.
- **Temporal and long-term land cover**: For water availability, a critical factor for livestock distribution, particularly in semi-arid and arid grazing systems, we included the *JRC Global Surface Water data set* (Pekel et al., 2017), which maps the location and temporal distribution of surface water bodies from 1984 onward using Landsat imagery. Furthermore, we prepared an ensemble global peatland extent data set using a compilation of peatland data sets (*i.e*., WRI Global Peatlands extent, PEATGRIDS and Global Peatlands Initiative) to characterize livestock grazing systems specific to wetland ecosystems (Hengl, 2024).
- **Temporal vegetation spectral indices**: Vegetation productivity, a key indicator of grazing capacity, was characterized by the Normalized Difference Vegetation Index (NDVI) using the MODIS product MOD13Q1 (16-day of temporal resolution and 250 m spatial resolution; Didan, 2021). We produced monthly cloud-free aggregates by computing the 50th percentile (median) of 16-day pixel values, providing a consistent representation of vegetation seasonality while maintaining sensitivity to changes in grazing resources over time.

To maximize the consistency and usability of all these spatial variables, we harmonized spatial and temporal resolution, imputed missing years and standardized variable formats across all raster layers. For data sets not available for the entire modeling period (2000–2022), we extended the series by replicating the values for the last years to subsequent years. In case of data sets with a higher spatial resolution (*e.g*., 250 m), the resampling to 1 km spatial resolution was implemented by the *average*, while coarser spatial resolution layers (*e.g*., 10 km) were resampled using the *cubicspline* method in GDAL (Qin, Zhan & Zhu, 2014). To ensure precise spatial alignment and minimize distortions, all data sets were reprojected to an equal-area coordinate system, specifically the Interrupted Goode Homolosine projection (ESRI:54052) using GDAL. This step was necessary to achieve correct co-registration between input features and livestock density values, as well as to ensure consistency in the final model predictions.

### Census data harmonization

Subnational livestock counts are critical for our modeling. Considering that most countries collect and publish their own agricultural statistics, we built a data harmonization pipeline to create a single, comprehensive database of cattle, horses, goats, sheep and buffaloes headcounts. The thematic, spatial, and temporal coverage of the final harmonized livestock census database is described in the result section. The pipeline included:

(A) **Data inventorying and downloading**: We first created an inventory of national agencies currently providing agricultural census data *via* publicly accessible hyperlinks. We prioritized repositories using open-access licensing (*e.g*., Creative Commons) and providing web services to enable data download. The download of the data occurred by each identified data source. The national agencies provided a wide range of file formats (*e.g*., Microsoft Excel and Access, CSV, PDF, HTML, custom DB formats) with few common standards and without general rules for associating statistical variables to each table. After download, all files were converted to CSV format and processed by the R-package tabshiftr (Ehrmann & Meyer, 2024) to reshape heterogeneous input tables based on schema descriptions organized for each data source (*e.g*., inconsistent livestock species terminology, and incompatible table layouts, such as headcount values distributed across varying row and column structures);
(B) **Polygon matching**: We prioritized matching the livestock data to official spatial data (from national statistical agencies) whenever available. If official data was not found, we used reliable and open-source alternatives: first, the OCHA’s Common Operational Data Sets—Administrative Boundaries (COD-AB) OCHA (Humanitarian Data Exchange, 2025), and second, the GADM 3.6 Database (Hijmans, 2019). This geographical prioritization reflects our preference for data sources with the highest authority over national territories. To ensure consistency and interoperability, we organized the data using the R-package arealDB (Ehrmann, Rümmler & Meyer, 2024a; Ehrmann, Seppelt & Meyer, 2020). The package helped us to link the livestock counts to the correct geographic polygon boundaries using text distance algorithms applied across multiple levels of administrative unit names (*e.g*., matching a municipality to a polygon name).
(C) **Ontology matching**: The different words related to livestock species were first translated to English and then matched with cattle, horses, goats, sheep, and buffalo using a ontology framework. We built our ontology with the R-package ontologics (Ehrmann & Rümmler, 2024) and mapped all external concepts to the harmonized LUCKINet land-use ontology (Ehrmann, Rümmler & Meyer, 2024b).
(D) **Data integration**: To increase the spatial and temporal coverage of our modeling, we supplemented our census database with existing harmonized data sets. Specifically, we integrated (i) a subset of the input census polygons used for producing GLW v4 (Wisser & Cinardi, 2024); (ii) the European census data compiled by Malek et al. (2024), which provides cattle and sheep headcounts for 2020 and 2010. For administrative units with overlapping data, we selected a single source to guarantee data consistency at each specific level. Priority was given first to our harmonized samples, followed by Malek et al. (2024), and lastly GLW v4.

### Preparation of potential land for livestock

To support the calculation of livestock densities and establish the modeling spatial domain, we prepared a time series of potential land for livestock (2000–2022) at 1 km spatial resolution. In our study, this is the assumed land with the primary use of livestock management, although we recognize that there are different livestock management systems and cultures in many countries. Livestock grazing can also occur in areas outside of grasslands, open shrublands, pastures, or croplands, which is not reflected in our potential land for livestock. The data set was derived by combining cultivated and natural/semi-natural grasslands (Parente et al., 2024b) and the extent of croplands (Potapov et al., 2022b), initially produced at 30 m and resampled to the target spatial resolution. We derived fractions of land mapped as cultivated grassland, natural/semi-natural grassland, and cropland, estimating the proportion of 30 m raster cells inside a 1 km raster cell (average as a resampling strategy). The integration considered the following thresholds to derive four global livestock production systems and the portion of land used by all of them together at 1 km spatial resolution:

(A) **Natural/semi-natural grassland systems** composed of raster cells where the fraction of natural/semi-natural is greater than cultivated grassland, and the fraction of cropland is 0–19%;
(B) **Cultivated grassland systems** composed of raster cells where the fraction of cultivated grassland is greater than natural/semi-natural grassland, and the fraction of cropland is 0–19%;
(C) **Mosaic cropland and grassland systems** composed of raster cells where the fraction of cultivated grassland is greater than 0%, and the fraction of cropland is 19–37%;
(D) **Cropland systems** composed of raster cells where the fraction of cultivated grassland is equal to 0%, and the fraction of cropland is 1–67%.

The previous integration thresholds for fraction of land were derived using the land system distribution map in 2015 from Venier-Cambron et al. (2024), where we estimated the spatial mean of 1 km cropland fractions inside the classes (i) *“Mosaic Crop and Grass—Low Intensity”*, (ii) *“Mosaic Crop and Grass—High Intensity”* and (iii) *“Cropland—Medium Intensity”*, and yielding values of 19%, 37% and 67%, respectively. Lastly, areas with extreme temperatures were considered not suitable for livestock production and masked according to the long-term MODIS land surface daytime temperature layers (2000–2021; Wan, Hook & Hulley, 2021). We masked all raster cells with less than −5 °C as long-term annual temperature or more than 48 °C as long-term annual maximum temperature, removing the majority of Tundra vegetated areas (Heijmans et al., 2022) in the North of Canada, Russia and Alaska (low temperatures), and core dry-land regions in Australia (Feng & Fu, 2013) and the Sahel region (high temperatures) from our livestock modeling. All final output raster products related to potential land for livestock are publicly available on Zenodo (Parente, Malek & Gonzalez Fischer, 2025).

### Calculation of livestock density

**Figure 2:**
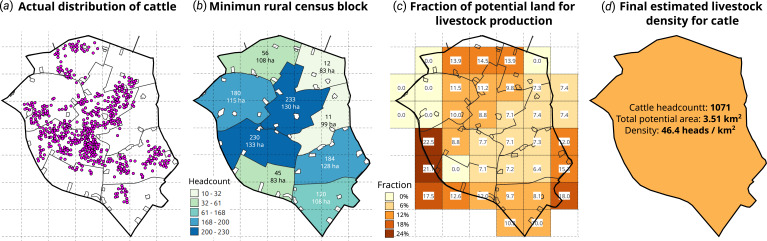
Schematic example of calculating livestock density for a census polygon in the Netherlands around Kampereiland–Municipality Kamper: (A) Actual distribution of cattle accessible only *via* a complete inventory of livestock at farm level, (B) aggregated headcount per minimum rural census blocks on specific time period (*e.g*., 1, 5 or 10 years), (C) fraction of potential land for livestock production based on existing grassland and cropland layers at 1 km resolution, and (D) final estimated livestock density for the census polygon, which is finally used by our modeling framework.

Usually, census surveys estimate livestock counts using statistical methods for a minimum rural census block (*i.e*., census microdata (DePaula & Veloso, 2025; Schultz, 2019)) since the actual distribution of livestock is accessible only through a complete inventory of livestock at the farm level (as illustrated in Figs. 2A and 2B). Livestock headcounts are then aggregated by administrative level (*e.g*., municipality, county, or country) and made publicly available by national agencies in various formats. To accurately calculate the density of the livestock using publicly available census headcounts, we used the potential land for the livestock production layer rather than relying on the total polygonal area (Fig. 2C). In this way, livestock densities were more realistically represented, reflecting the actual land available for livestock species rather than including non-agricultural areas within the administrative or geographical boundaries. For estimating total areas, all raster layers were reprojected to the Interrupted Goode Homolosine projection, an equal-area composite projection, and for each census polygon, all 1 km fraction raster cells inside the polygon boundaries were added together. The livestock density values were then calculated for each polygon, each livestock species and each year using the total headcount and total potential area for livestock production (Fig. 2D).

### Spatiotemporal zonal statistics

We generated the feature set for the areal regression models by first computing the annual mean at polygonal level for all 128 raster layers in all administrative units of the harmonized census database. This calculation only included raster cells that were within the polygon and had at least 1% potential land for livestock production in a given year. The same criteria were applied when calculating the density of the livestock. Since the harmonized annual raster layers (*e.g*., MODIS vegetation index, precipitation) span 2000–2022, we matched their year with the census polygon’s reference year to ensure consistency in the spatiotemporal mean. For static and long-term raster layers, the spatial mean was replicated across all years. Lastly, we created a sample data set using these annual livestock density values and the calculated spatiotemporal means.

### Sample filtering, splitting and weighting

To prepare the sample data set for our modeling approach, we (i) filtered sample outliers (*i.e*., 98th percentile), (ii) imputed zero density values (*i.e*., 2nd percentile), (iii) split them into training, calibration and testing sets, and (iv) derived sample weights for model benchmarking. All these steps were applied independently for each livestock species, considering all samples with density values greater than zero (several census data sets use zeros instead of empty or NA for non-reported livestock headcount). As density values were derived using two very different data sets (*e.g*., headcount from census and potential area of remote sensing), we identified unrealistically high-density values (*e.g*., 5,000 heads per km^2^) and low-density values (*e.g*., 0.01 heads per km^2^) in various samples. High-density values were systematically removed by using an outlier threshold based on the 98th percentile (estimated using all samples for a specific livestock species), while low-density values were converted to actual zero headcount according to the 2nd percentile. Due to the ambiguity between true zero headcounts and missing (NA) values within the source data, these records could not be reliably distinguished and were omitted from the analysis. The final number of spatiotemporal samples used by our modeling approach is presented in Table 1.

**Figure 3:**
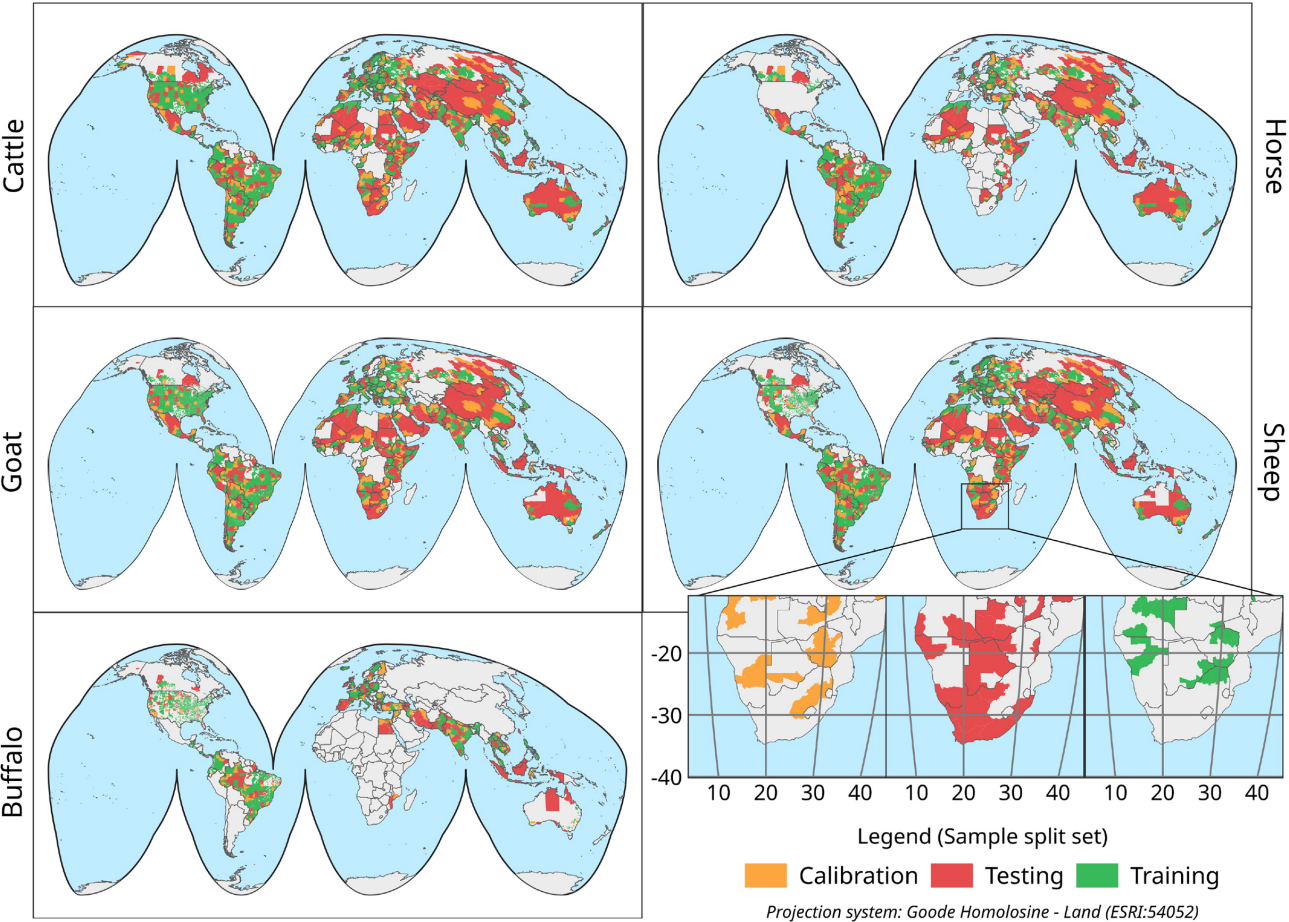
Spatial distribution of subnational harmonized livestock census database classified into training, calibration and testing sets. The final models were fitted using training and calibration sets, while the testing set was used to derive the technical validation.

The samples were then randomly split into three sets, with 80% for training and 10% for calibration and testing, each, while retaining all annual values of a polygon in the same set. This approach used a blocking strategy based on regular tile systems (200 × 200 km) to reduce the spatial autocorrelation in our modeling, keeping all polygons intersecting with a specific tile only in one split set (Fig. 3). The sample weights were derived according to Eq. (1) and rescaled to 0–1 by min-max normalization. Deriving sample weights that are inversely proportional to the area of a census polygon decreases the importance of large polygons in the training process, mitigating the limitation that areal regression is sensitive to the size of the input polygons.

(1) $$W = {1 \over {\sqrt {{{AC} \over \pi }} }}$$

(2) $$W^{\prime} = {{W - {W_{\min }}} \over {{W_{\max }} - {W_{\min }}}}$$

where:

*AC* = Area of the minimum bounding circle for a census polygon

*W* = Sample weight

*W*′ = Normalized sample weight.

### Model training and optimization

Model training and optimization were run independently for each livestock species, considering the sample data set prepared and filtered by the previous steps. For model benchmarking, we compared two ML models, Quantile Regression Forest (QRF) (Mathlouthi, Fredette & Larocque, 2015) and Gradient Boosting Trees (GBT) (So, 2024) considering different combinations of (i) transformed (BoxCox transformation (Atkinson, Riani & Corbellini, 2021)) and non-transformed livestock densities, and (ii) weighted and non-weighted samples. The optimization used the calibration set (*i.e*., 10% of samples) to first select the most important features by Recursive Feature Elimination (RFE) (Demarchi et al., 2020), and later search/tune the model hyper-parameters by Successive Halving—SH (Jamieson & Talwalkar, 2016), maximizing the accuracy performance of the models.

The implemented RFE considered a standard Random Forest model with 50 trees, Poisson criterion as a measurement of the quality of the split, and default values of other hyper-parameters; fitted using the scikit-learn library (Shaharum et al., 2018). The decision to use the Poisson criterion was based on the fact that many census data sets, including livestock densities, follow the Poisson distribution, which is very skewed and with the majority of values concentrated close to zero. For each RFE iteration, the 12 least important features were removed (according to Gini importance), resulting in 50 features as the final selection (*i.e*., about 40% of the total number of features).

The selected features were then used to run SH using scikit-learn (Shaharum et al., 2018). Here we specifically used the D^2^ regression score metric (O’Brien, 2016), based on the five-fold spatial blocking cross-validation (note: blocking is implemented using county, or municipality name) to iteratively assess different combinations of hyper-parameters candidates bounded by a customized search space. The assessment started with about 5,000 samples, selecting the best candidates (*i.e*., dropping half of the less accurate candidates) and doubling the number of samples per iteration until the full set of calibration samples was reached. Once the last iteration was done, the hyper-parameters with the best value of D^2^ (*i.e*., the highest D^2^) were selected for each ML model. Specifically for GBT, an early stop strategy was used (in half of the calibration set) to find the best number of trees and minimize the risk of over-fitting (Raskutti, Wainwright & Yu, 2014). The optimization found 10 sets of hyper-parameters (*i.e*., two ML models and five livestock species), which were used to train the final models with 90% of total number of samples (*i.e*., train and calibration set combined; Fig. 3). The most accurate model (according to the testing set—10% of the samples) was selected to produce final global predictions of the livestock species distributions.

### Model benchmark and technical validation

For model benchmarking and technical validation (final selected models), the testing set (*i.e*., 10% of the samples) was used to calculate D^2^ regression score (see Eq. (4)) (O’Brien, 2016), RMSE, and CCC (Eq. (5)) (Steichen & Cox, 2002). Although not very common in most regression modeling studies, we believe that D^2^ metric is the most suitable metric to evaluate skewed distributions (*e.g*., Poisson distribution), which is the case for the livestock densities models implemented in this study:

(3) $${D^{2}} = 1 - {{{D_{\rm {model} }}} \over {{D_{\rm {null} }}}}$$

(4) $$D({y_{i}},{\hat y_{i}}) = {1 \over n}\sum\limits_{i = 1}^n {\left[ {{y_{i}}\log {{{y_{i}}} \over {{{\hat y}_i}}} - ({y_{i}} - {{\hat y}_i})} \right]}$$

(5) $$CCC = {\displaystyle{2 \cdot {\mathrm{Cov}} (y,{\hat{\hat{y}}})} \over {{{\rm {Var}} (y) + {\mathrm{Var}} ({\hat{\hat{y}}}) + ({\bar{y}} - {{\bar{\hat y}}})^2}}}$$

where:

${D_{{{\mathrm{model}}}}}$ = Mean Poisson deviance of the fitted model

${D_{{{\mathrm{null}}}}}$ = Mean Poisson deviance of the null model, which predicts the mean $\bar y$ of the observed values.

${{\mathrm{Cov}}}(y,\hat y)$ = Covariance between observed and predicted values

${{\mathrm{Var}}}(y)$ = Variance of the observed values

${{\mathrm{Var}}}(\hat y)$ = Variance of predicted values

${y_{i}}$ = Observed value

${\hat y_{i}}$ = Predicted value

$n$ = Number of samples.

### Global predictions

The best ML models were used to make global predictions for each livestock species annually (2000–2022). The predicted density values were estimated in Interrupted Goode Homolosine projection (equal-area composite projection; ESRI:54052) and then multiplied by the fraction of potential land for livestock production (which establishes our modeling spatial domain), resulting in global distribution for cattle, goats, sheep and horses at 1 km spatial resolution. The prediction intervals per raster cell (2.5th and 97.5th percentiles—95% probability distribution) were estimated through the individual predicted values retrieved from the final ensemble tree models.

The final mosaics were made available as Cloud-Optimized GeoTIFFs with three raster outputs per year per species (lower, mean, and upper prediction intervals). In total, we produced 690 global raster files: five (5) livestock species $\times$ 23 years $\times$ three (3) livestock densities (lower, mean and upper) $\times$ two (2) headcount (raw and FAOSTAT-adjusted), which is in total about 65 GB of data. The total time needed to fine-tune and train all models was 4 h, and total prediction time was 24 h using a single CPU server with 1 TB of RAM and 96 processing threads. Note that once the spatiotemporal models are fitted, we can continue to predict livestock densities for more recent years (*e.g*., 2023 and onward) assuming that the new values of feature layers will be available.

### Calculation of headcount

A second set of global distribution raster layers was generated through a linear adjustment factor (Eq. (6)), aligning the number of livestock species on the national scale, as reported by FAOSTAT (version 2024.06; https://www.fao.org/faostat/en/#data), with the predicted raster cell values for each specific country. This adjustment was applied individually to each country, ensuring that the adjusted values accurately reflected the national totals. In practice, this approach guaranteed that the sum of the raster cell values in each corrected map closely matched the livestock statistics provided by FAOSTAT for each country, thereby improving consistency with national totals and usability for applications requiring FAOStat alignment:

(6) $$af = {{\sum\nolimits_{i = 1}^n {{{\hat y}_i}} } \over {{y_{\rm faostat}}}}$$

where:

${y_{\rm faostat}}$ = Total number of livestock species according to FAOSTAT

${\hat y_{i}}$ = Predicted number of species per raster cell

$n$ = Number of predicted raster cells in the country

## Results

### Harmonized livestock census database

**Figure 4:**
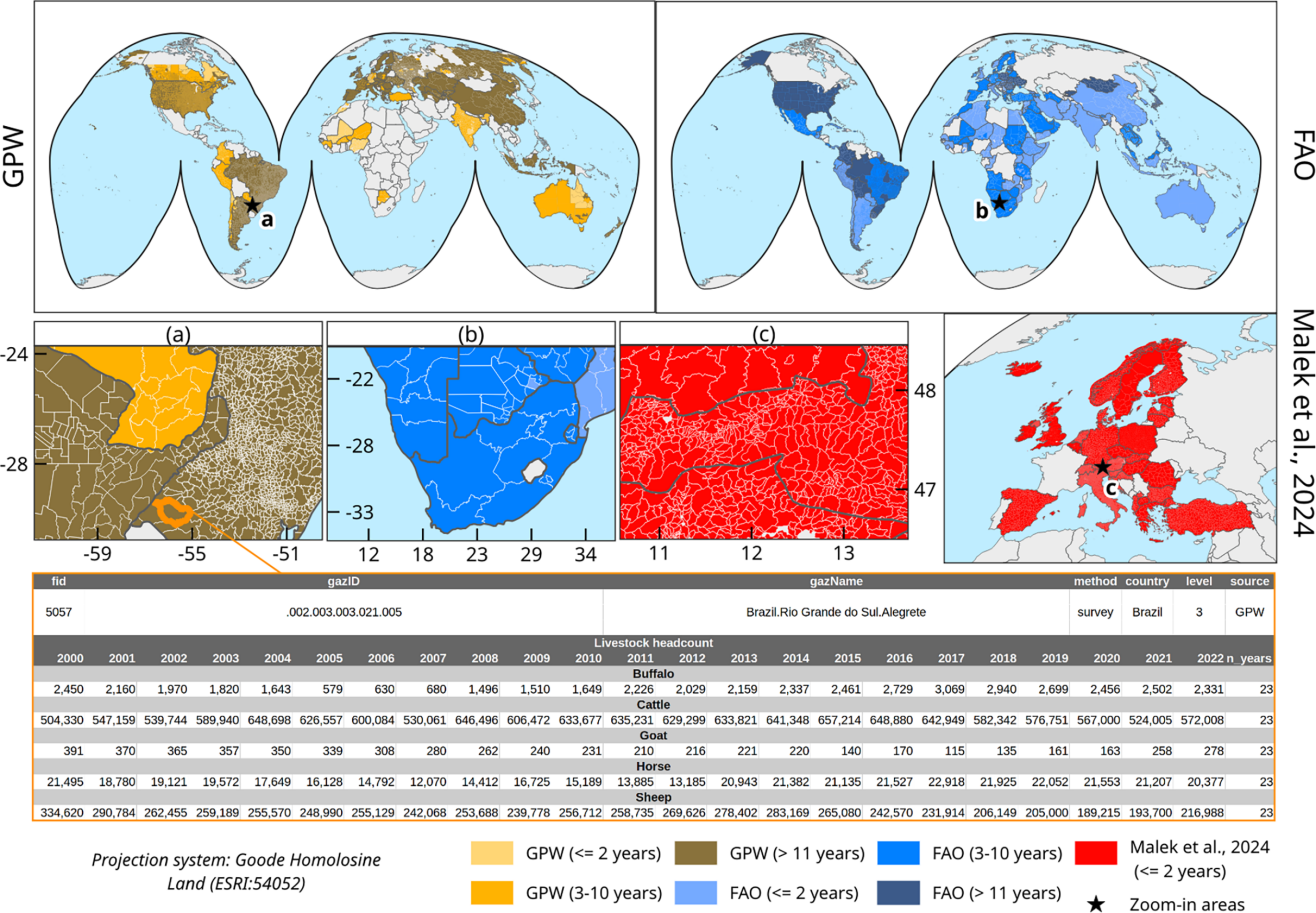
Spatial distribution of subnational livestock census data harmonized in this study, including unique code and name of the administrative units, country name and multi-year headcounts for cattle, horse, goat, sheep and buffalo. The database is composed of (A) *GPW* (data collected by this study), (B) *FAO* (subset of input polygons used for producing GLW v4 (Wisser & Cinardi, 2024)), and (C) European census data compiled by Malek et al. (2024).

Our final livestock census database includes 55,336 administrative units, where 20,032 units were harmonized by this study, and 22,632 and 12,672 units integrated from FAO Wisser & Cinardi (2024) and Malek et al. (2024), respectively (Fig. 4). Covering 147 countries, the database is composed of administrative levels 2 (state), 3 (municipality), and 4+ (district and subdistrict), which has a mean polygon size of 2,907 km^2^; SD = 21,463. The units provided by Malek et al. (2024) have the smallest polygon area (248 km^2^; SD = 1,210), while FAO has the largest polygon area (5,562 km^2^; SD = 27,028—Table S1).

Temporal coverage for livestock counts is irregular and varies significantly across countries and species. The number of countries with more than 20 years of data differs greatly by species: 20 countries for cattle (*e.g*., Austria, Brazil, China, Indonesia, New Zealand, Poland, the United States); 13 countries for buffaloes (*e.g*., Belgium, Brazil, Czechia, Finland, Poland, Portugal); 10 countries for sheep (*e.g*., Brazil, China, France, Italy, New Zealand, Romania, United Kingdom and Northern Ireland, the United States); nine countries for goats (*e.g*., Brazil, China, France, Indonesia, Portugal, Romania, Spain); and three countries for horses (*i.e*., Brazil, China, Indonesia). In the database, France, Italy, Portugal, and Spain have fewer than 20 years of headcount records for cattle, buffaloes, goats, and sheep, but no records for horses. Various countries have counts only for 1 year, including: 32 countries for sheep (*e.g*., Afghanistan, England, South Korea); 27 countries for goats (*e.g*., Bolivia, Guinea, Switzerland) and horses (*e.g*., Costa Rica, Haiti, Tunisia); 24 countries for cattle (*e.g*., Algeria, Nepal, the Netherlands); 11 countries for buffaloes (*e.g*., Armenia, Costa Rica, Mozambique). Only Brazil and Indonesia have annual counts for all five livestock species over all years (2000—2023), although Brazil provides data at level 3 and Indonesia at level 2.

Most countries in Asia and Africa have only level 2 data (24 and 22 countries, respectively). In Africa, 12 countries partially have level 3 and 4+ data, where the highest data availability can be found in Kenya, Ethiopia, and Niger (469, 209 and 107 administrative units each). In Asia, 13 countries partially have level 3 and 4+ data, where Russia, Turkey and India (1,739, 982 and 659 administrative units each) are the most representative ones. In Oceania, most of the data are level 2 and 3, while in Europe, North America, and South America, most of the data are level 3 and 4+. All polygon inputs (raw data) and harmonized outputs are publicly available on Zenodo (https://doi.org/10.5281/zenodo.17665040; Parente et al., 2024a).

### Modeling benchmark and technical validation

The accuracy assessment benchmark shows that the Random Forest (RF) outperforms the Gradient Boosting Trees (GBT) for all species and modeling strategies (*i.e*., weighted samples and BoxCox transformation; see Table 2). RF without data transformation and weighted samples shows the highest CCC values for modeling cattle, sheep, and goat densities. For modeling horses and buffaloes, the strategy using the BoxCox transformation and weighted samples shows the best performance on the same accuracy metric. The same strategy showed better performance in terms of D^2^ and RMSE for modeling cattle, horses, and buffaloes. RF without data transformation and weights show the most consistent result for sheep modeling, with the highest values for all three accuracy metrics in all modeling strategies. GBT with data transformation and weights presented the worst performance for all livestock species.

**Table 2:** Benchmark results for machine learning modeling (random forest (RF) and Gradient-Descent Trees (GBT)) of livestock densities, considering different combinations of (i) transformed (BoxCox transformation), and non-transformed target variable, and (ii) weighted and non-weighted samples. The best models and highest accuracy metric values are highlighted in bold.

Livestock species	Modeling strategy	CCC	${D^{2}}$	RMSE
Cattle	GBT	0.502	0.438	111.501
	GBT/BoxCox trans.	0.498	0.423	111.633
	GBT/BoxCox trans./weighted samples	0.149	0.021	135.056
	GBT/weighted samples	0.409	0.333	118.774
	**RF**	**0.603**	0.531	104.586
	**RF/BoxCox trans.**	0.591	**0.545**	**103.675**
	RF/BoxCox trans./weighted samples	0.549	0.508	106.494
	RF/weighted samples	0.574	0.501	106.507
Horse	GBT	0.531	0.423	6.145
	GBT/BoxCox trans.	0.477	0.387	6.183
	GBT/BoxCox trans./weighted samples	0.308	0.07	6.852
	GBT/weighted samples	0.401	0.303	6.432
	RF	0.547	0.454	6.06
	**RF/BoxCox trans.**	0.548	**0.495**	**5.9**
	**RF/BoxCox trans./weighted samples**	**0.566**	0.481	5.929
	RF/weighted samples	0.54	0.438	6.108
Sheep	GBT	0.567	0.566	69.95
	GBT/BoxCox trans.	0.48	0.494	73.471
	GBT/BoxCox trans./weighted samples	0.315	0.188	80.931
	GBT/weighted samples	0.474	0.42	75.59
	**RF**	**0.622**	**0.621**	**67.575**
	RF/BoxCox trans.	0.606	0.606	67.82
	RF/BoxCox trans./weighted samples	0.58	0.586	68.777
	RF/weighted samples	0.6	0.589	68.754
Goat	GBT	0.546	0.59	66.3
	GBT/BoxCox trans.	0.452	0.538	68.239
	GBT/BoxCox trans./weighted samples	0.268	−0.072	77.173
	GBT/weighted samples	0.417	0.437	71.807
	**RF**	**0.598**	0.645	**64.09**
	RF/BoxCox trans.	0.591	0.617	66.831
	**RF/BoxCox trans./weighted samples**	0.596	**0.647**	64.392
	RF/weighted samples	0.553	0.604	65.543
Buffalo	GBT	0.631	0.695	31.951
	GBT/BoxCox trans.	0.281	0.374	39.05
	GBT/BoxCox trans./weighted samples	0.129	0.118	42.436
	GBT/weighted samples	0.275	−0.118	43.511
	RF	0.689	0.724	30.369
	**RF/BoxCox trans.**	**0.697**	0.736	**29.279**
	**RF/BoxCox trans./weighted samples**	0.671	**0.739**	30.003
	RF/weighted samples	0.676	0.718	30.136

To select the final modeling strategy, we prioritized the performance of the CCC, which is a metric designed to measure the agreement between predicted and observed values, capturing both the scatter of the data around the $y = x$ line (precision) and the distance between the mean prediction and the mean observation. This aspect is particularly relevant in our modeling, once we implement areal regression at the polygonal level (using mean feature values) and produce spatial predictions at the raster cell level (1 km resolution). Given that the CCC performance was comparable (or similar) without the Box-Cox transformation, we prioritized the simpler, untransformed modeling strategy, given the intrinsic differences between the modeling and prediction scales, and the potential risk of smoothing out predicted densities at raster cell level. Thus, we finally selected RF without transformation and weights to produce global predictions for all species.

**Figure 5:**
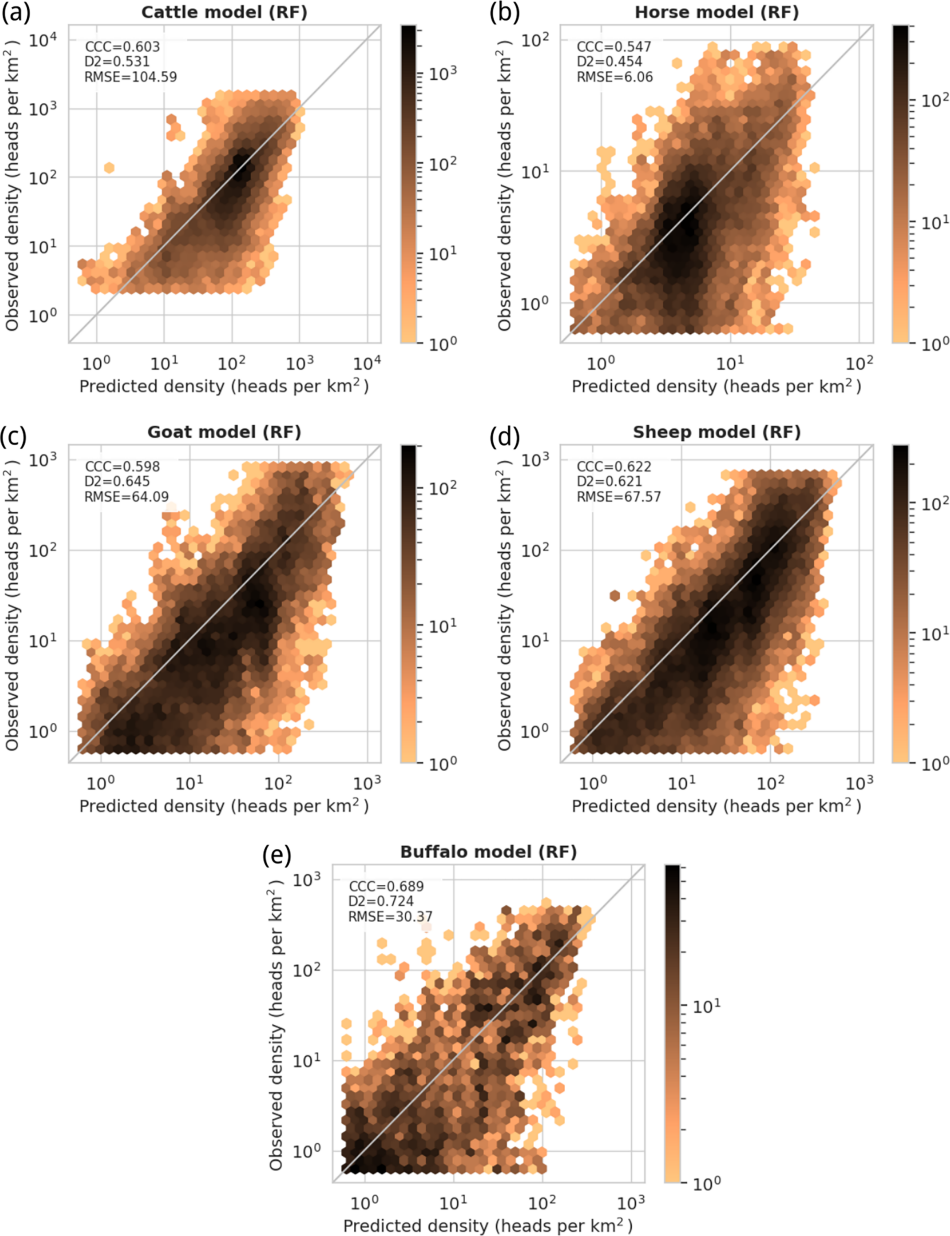
Technical validation for final selected models based on the testing samples set for (A) cattle, (B) horse, (C) goat, (D) sheep and (E) buffalo.

Analyzing the final selected models, the buffalo density model is the most accurate (*i.e*., CCC 0.689 and D^2^ 0.724; Fig. 5E), while the horse density model is the least accurate (*i.e*., CCC 0.547 and D^2^ 0.454; Fig. 5B). RMSEs for cattle, buffaloes and horses are 104.59, 30.37, and 6.06, respectively (Figs. 5A, 5B, and 5E). The predicted distributions of the goat and sheep models are similar; however, the goat model shows lower RMSE (*i.e*., 64.09—Fig. 5C) compared to sheep (*i.e*., 67.57—Fig. 5D). All models seem to overestimate low values (*i.e*., less than 10 heads per km^2^) and underestimate high values (*i.e*., around 100 heads per km^2^ for horses; 500 heads per km^2^ for buffaloes and 1,000 heads per km^2^ for cattle, goats and sheep).

### Key explanatory variables of livestock density

**Figure 6:**
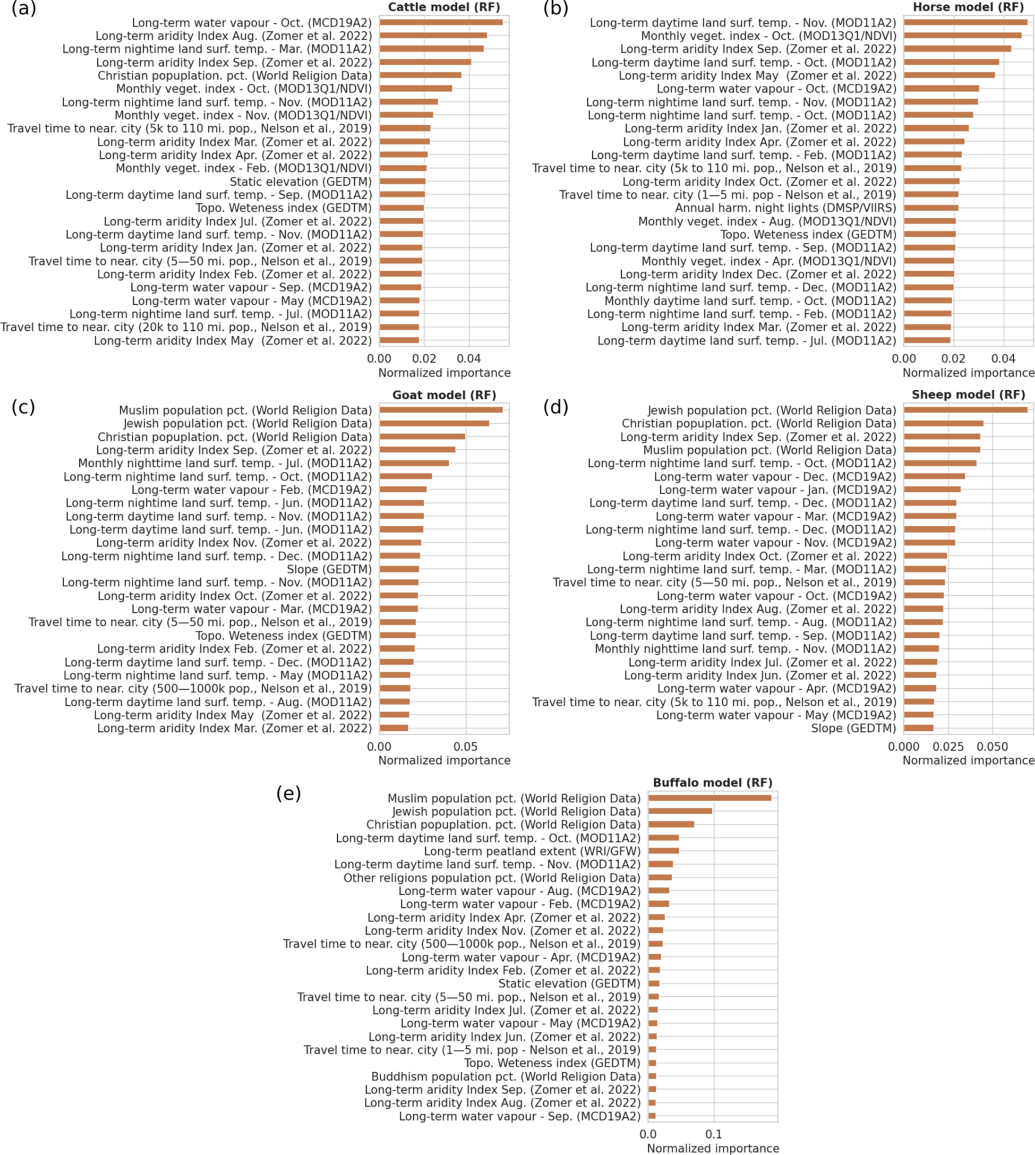
The 25 most important features for modeling global distribution of (A) cattle, (B) horse, (C) goat, (D) sheep and (E) buffaloes based on the random forest algorithm.

Based on the RF models fitted, climate and socio-economic layers appear to be the most important variables for predicting livestock densities, according to the feature importance analysis (Fig. 6). Across all models, the top 10 features frequently include long-term water vapor (MCD19A2), the aridity index, and the daytime/nighttime land surface temperature (MOD11A2). Additionally, travel time to the nearest cities (of various population sizes) consistently ranks among the top 20 most important features. Population’s religion (based on the World Religion Data) is the most important single feature across the models for goats (Fig. 6C), sheep (Fig. 6D), and buffaloes (Fig. 6E). For cattle and horse densities, the monthly vegetation index (MOD13Q1 NDVI) is particularly important, with different months consistently ranking among the top 25 features (Figs. 6A and 6B). In terms of topography, slope (GEDTM) is a significant feature, appearing in the top 25 for both goats (Fig. 6C) and sheep (Fig. 6D), while the topographic wetness index (GEDTM) is highlighted for modeling cattle (Fig. 6A), horses (Fig. 6B) and buffaloes (Fig. 6E). The peatland extent is one of the most important features for modeling the density of buffaloes (Fig. 6E). Light at night is an important feature specifically for horses in our modeling (Fig. 6B). It should be noted that annual GDP and HDI were not among the 25 most important features for modeling any of the livestock densities. A significant difference exists in the distribution of feature importance: the buffalo model’s values are more skewed toward its top five features, indicating a high concentration of predictive power, whereas the importance values for the other models are more evenly distributed across their top 25 explanatory features (Fig. 6).

**Figure 7:**
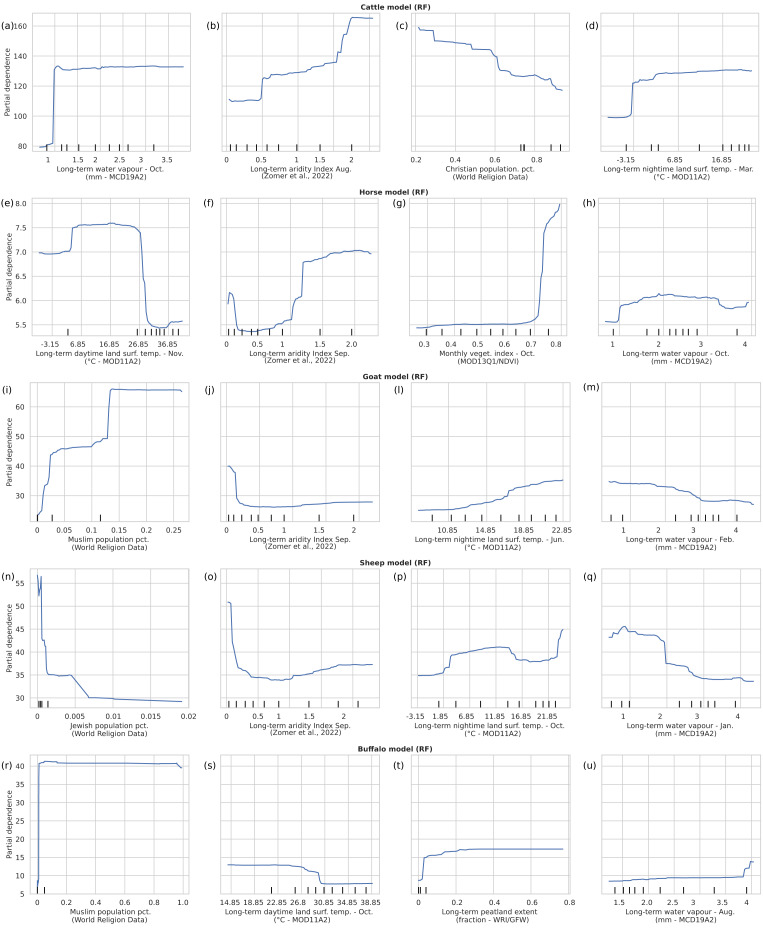
Partial dependence analysis for the final random forest models considering the four most important features for cattle (A–D), horse (E–H), goat (I–M), sheep (N–Q) and buffalo (R–U). Note that the correlations presented here rely on the input census data which are often irregular, incomplete and varies widely by regions.

Partial Dependence Analysis (PDA) (Hastie et al., 2009) was used to understand the marginal effect of the most important features on the modeled livestock densities (Fig. 7). We are interested in understanding whether the relationship between the target and a specific feature is linear, monotonic, or more complex. Most of the features affect the densities of each livestock species in a different way. Taking into account long-term water vapor, the densities of cattle and horses increase dramatically around one millimeter in October with further stabilization (Figs. 7A and 7H), while for goats and sheep a monotonic decreasing trend is visible for water vapor ranging from 1 to 4 mm (Figs. 7M and 7Q). For buffaloes, water vapor sharply increases densities around 4 mm (Fig. 7U), although this effect is smaller compared to cattle and horses. Horse, goat and sheep densities decrease sharply from 0 to 0.5 m according to the long-term aridity index in September (Figs. 7F, 7J, and 7O); however, densities stabilize later for goat and sheep, while they increase back after 1 mm for horses. Analyzing the land surface temperature, cattle densities sharply increase after −3 °C at night time in March (Fig. 7D), while buffalo densities sharply decrease after 30 °C during the daytime in October (Fig. 7S). For horses, densities are higher between 6.85 °C and 26.85 °C during the daytime on November (Fig. 7E). Densities of goats and sheep monotonically increase according to temperature (Figs. 7L and 7P). Regarding religious populations, there are distinct relationships with livestock density. Densities of goats and buffaloes increase sharply as the proportion of Muslim people in a population rises (Figs. 7I and 7R). Conversely, densities of sheep appear to be highest in countries where there is 0% adherence to Judaism (Fig. 7N). One possible explanation is that many Jewish and Muslim people do not eat pork, so it is expected that goats, sheep and buffaloes replace pigs in their diets. Cattle densities show a consistent decrease as the proportion of the Christian population increases, specifically within the range of 20% to 80% (Fig. 7C). Separately, environmental factors also play a role: increases in the vegetation index seem to correspond to higher densities of horses (Fig. 7G), while increases in the extent of peatland are associated with higher densities of buffaloes (Fig. 7T). This illustrates that relationships for different species of livestock have unique patterns, and these can often be complex.

### Annual layers of global livestock distribution

**Figure 8:**
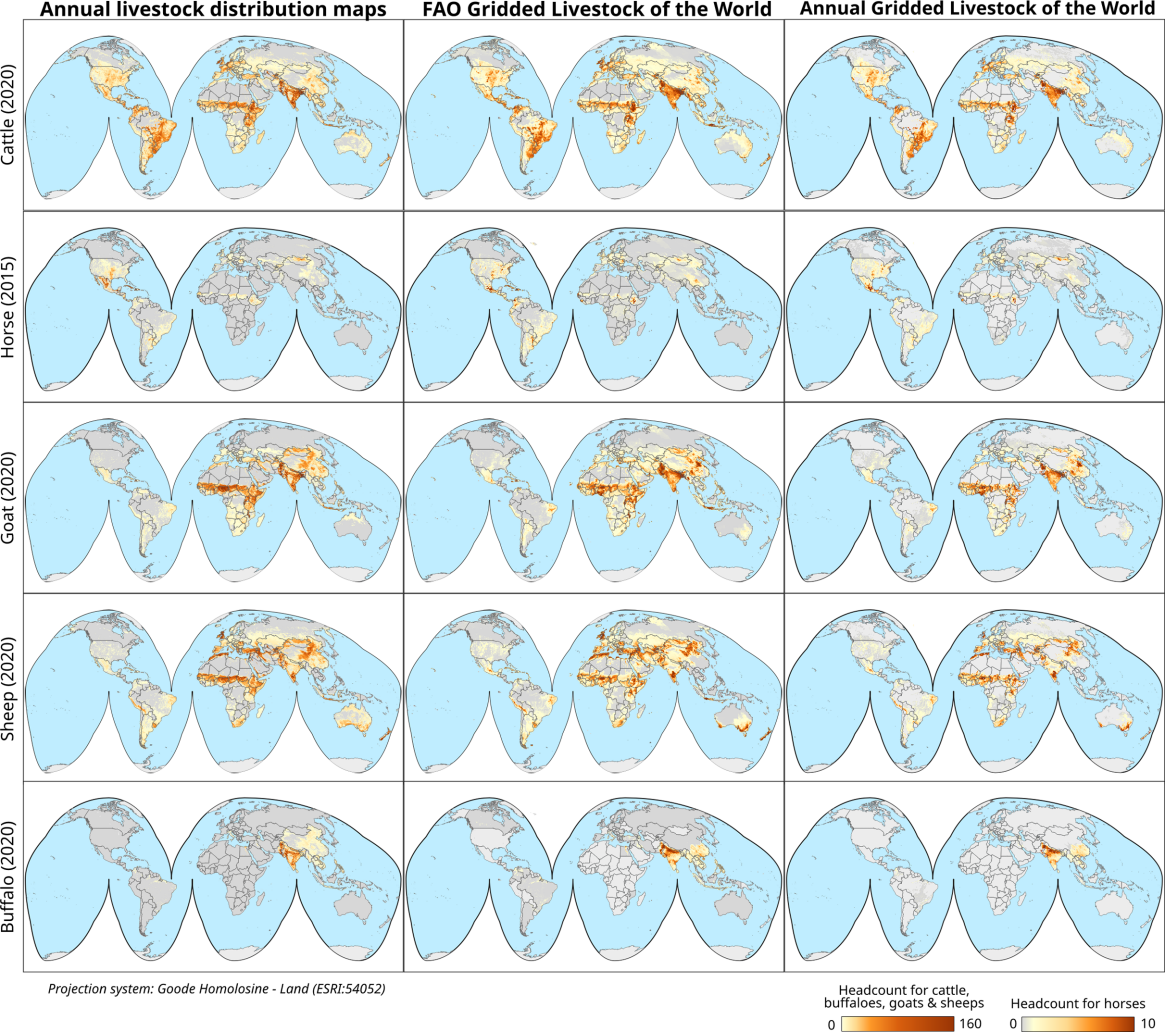
Global distribution layers for cattle, goats, sheep, horses and buffaloes compared to FAO gridded livestock of world (v3 for horses and v4 for all other species; Wisser & Cinardi, 2024) and annual gridded livestock of world (Du et al., 2025) in 2020 and 2015.

**Figure 9:**
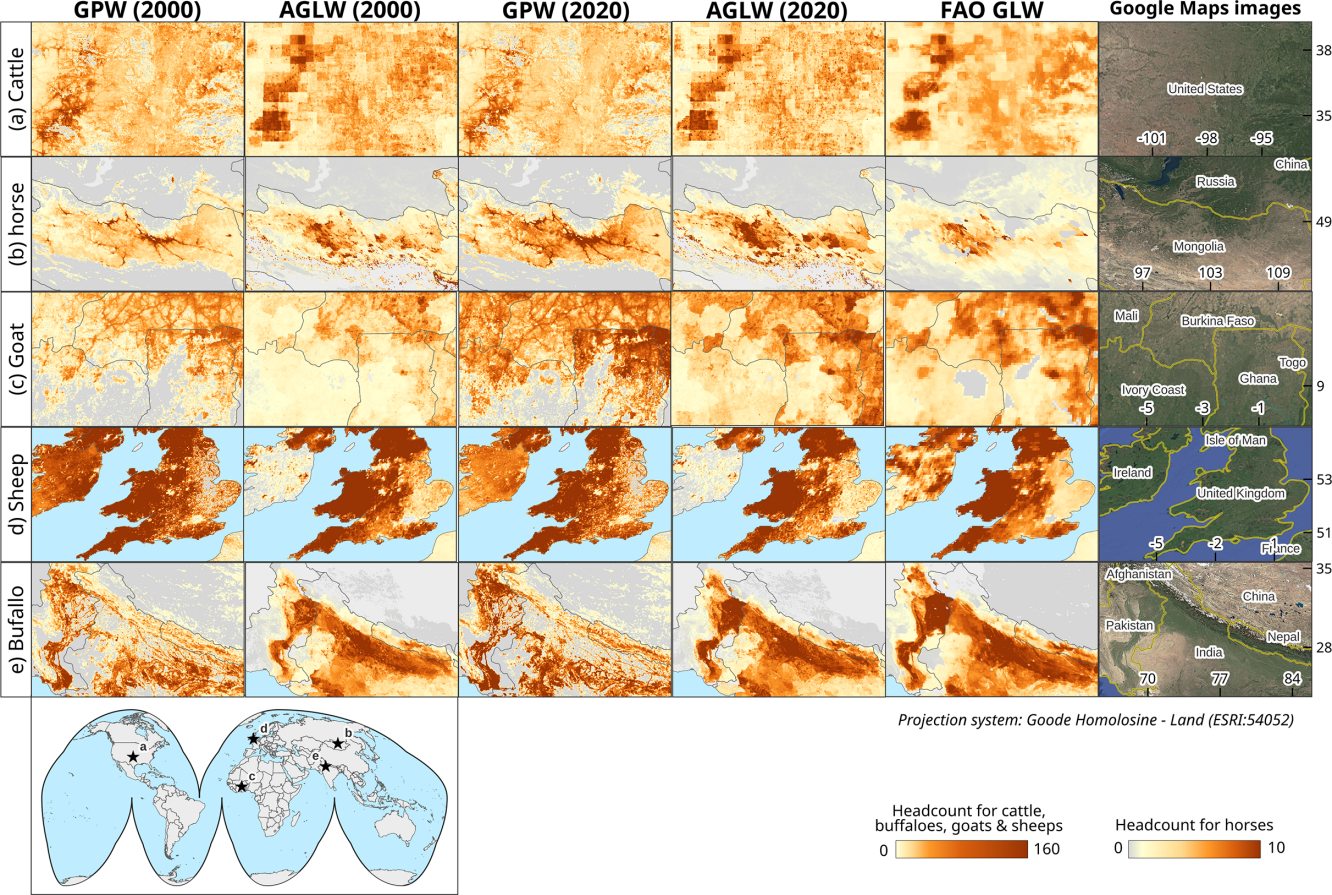
Comparison of our predicted annual livestock headcounts with FAO gridded livestock of world (Wisser & Cinardi (2024)—v3 for horses and v4 for all other species) and annual gridded livestock of world (Du et al., 2025) for different livestock species in the United States (A), Mongolia (B), West Africa (C), United Kingdom (D) and North of India (E).

**Figure 10:**
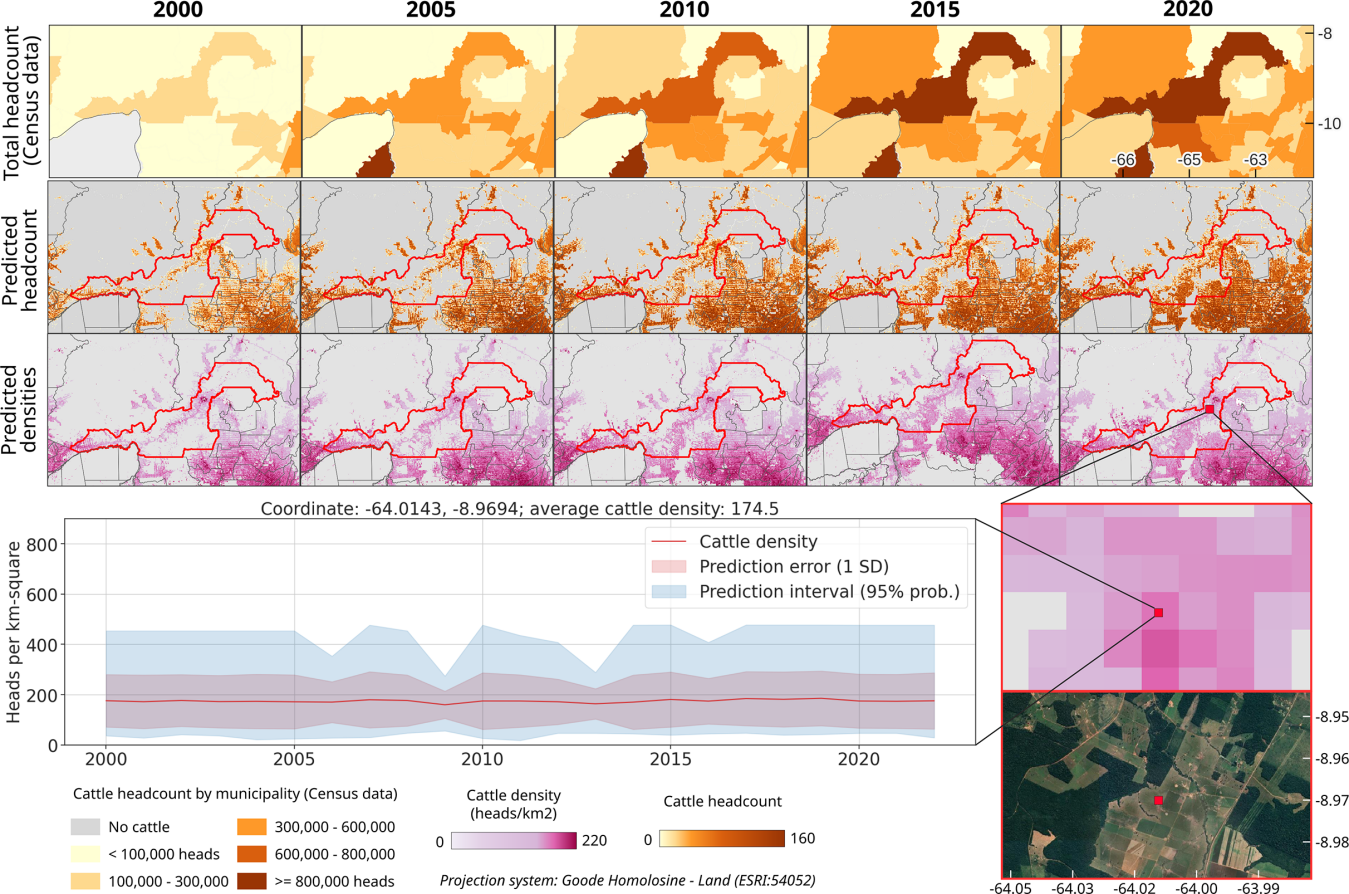
Examples of produced predictions (time-series) for cattle, in comparison with the original census data for the state of Rondônia in Brazil across 20 years. Capital of state, Porto Velho, is highlighted in red and the time series plot shows prediction intervals, prediction error and predicted mean for cattle density according to our modeling framework. Note that our 1 km livestock predictions do not exhibit the polygon structure found in the census data. This is because the majority of the features we use are Earth Observation (EO) data, which are independent of the original census boundaries.

Our global predictions cover the period of 2000 to 2022, on an annual basis, providing livestock headcounts of cattle, horses, goats, sheep and buffaloes, per raster cell at 1 km spatial resolution, as shown in Figs. 8 and 9. The output layers seem to capture changes in land cover and land use, once they incorporated the annual layers of potential land for livestock production. This is visible on tropical deforestation frontiers, such as Indonesia, the Paraguayan Chaco, and the Brazilian Amazon and Cerrado biomes (Fig. 10). Because we have adjusted the headcounts to match the FAOSTAT national values, the country borders are visible in some areas (*e.g*., Ireland, Mongolia, Mozambique, the Netherlands, and the United States). Specifically about horses and buffaloes distributions, layers show large areas with zero counts and temporal inconsistencies in some of the mapped countries (*e.g*., Belgium, Mongolia, the United States), which might be explained by a mismatch between FAOSTAT and our predictions (based on subnational livestock census data). In Mongolia, for example, the reported FAOSTAT horse headcounts vary significantly over the years (*e.g*., about 3.1, 1.9 and 4.8 million heads in 2000, 2010 and 2022, respectively), and these fluctuations are reflected in our output layers. Data gaps in FAOSTAT are also reflected in our output layers, such as not-reported horse headcounts for Italy from 2017 to 2022 and goat headcounts for Ireland from 2020 to 2021.

### Comparison with existing livestock products

Compared to FAO Gridded Livestock of the World (GLW; Wisser & Cinardi, 2024) and Annual Gridded Livestock of the World (AGLW; Du et al., 2025), in general, our output layers show similar spatial patterns at global scale for all livestock species (Fig. 9). The three products present hotspots of cattle (*i.e*., greater than 120 heads) in Latin America (*e.g*., Argentina, Brazil, Colombia, Dominican Republic, Mexico, Nicaragua, and Venezuela), North America (*e.g*., Canada and the United States; Fig. 9A), Sahel region (*e.g*., Burkina Faso, Ethiopia, Senegal, and South Sudan), East Africa (*e.g*., Kenya, Uganda, and Tanzania), Europe (*e.g*., England, France, Ireland, and the Netherlands), East Asia (*e.g*., China, Myanmar, and India), Australia and New Zealand. In India, the headcount numbers are more clustered geographically in our predictions, while in countries such as Australia, Canada, China, Ethiopia, Italy, Madagascar, and the United States, they are more widely distributed across the national territory. Specifically in Australia, our predictions allocated more cattle in the north of the country, while FAO GLW and AGLW layers show higher densities in the west.

Goats and sheep have similar spatial patterns with hotspots (*i.e*., greater than 100 heads) in the Sahel region (*e.g*., Chad, Ghana, Kenya, Senegal, and Togo; Fig. 9C), the Middle East (*e.g*., Iran, Lebanon, Syria, and Yemen), East Asia (*e.g*., China, India, Indonesia, Mongolia, and Pakistan) in all products. Specifically for sheep, the three products show hotspots in South America (*e.g*., Bolivia, Peru, and Uruguay), Europe (*e.g*., Greece, Italy, Spain, Romania, and United Kingdom; Fig. 9D), South of Australia, and New Zealand. Compared to GLW and AGLW, our predictions show goats and sheep more spatially distributed across national territories in Australia, Brazil, China, France, India, and Mexico. Horses and buffaloes exhibit a lower density compared to the other livestock species we mapped. Areas with high horse counts (*i.e*., greater than six heads) consistently appear across the three products in: Argentina, Chad, China, Cuba, Ethiopia, Indonesia, Kazakhstan, Kyrgyzstan, Mexico, Mongolia, Portugal, Senegal, the United Kingdom, and the United States (Fig. 9B). Regarding buffaloes, the hotspots (*i.e*., greater than 100 heads) are mostly concentrated in East Asia, specifically in: Bangladesh, China, India, India, Myanmar, Pakistan, the Philippines (Fig. 9E).

## Discussion

### Key discoveries and general implications

We have produced global distribution layers of livestock densities and headcounts for cattle, horses, sheep, goats and buffaloes at 1 km annually for the period 2000–2022 (Fig. 10). As key inputs, we used the largest available compilation of subnational livestock census data, covering approximately 86% of the potential land for livestock production layer at multiple levels of administrative divisions, during at least 1 year in our modeling horizon (Table S1). Although this constitutes a major improvement in the spatial and temporal coverage of input data over the current best available livestock products (FAO GLW (Wisser & Cinardi, 2024) and AGLW (Du et al., 2025)), typical patterns of irregular reporting and data gaps in the Global South remain visible in our harmonized census data, especially in parts of Africa and Asia (Fig. 4). However, our technical validation (based on the testing set) shows that our predictions accurately match livestock density patterns at subnational administrative-unit level (CCC = 0.603/0.547/0.598/0.622/0.689 and RMSE = 104.59/6.06/64.09/67.57/30.37 for cattle/horses/sheep/goats/buffaloes in multi-level administrative divisions with a mean polygon area of 2,907 km^2^, SD = 21,463; see Fig. 4). At these spatial scales, the presented data set has the potential to support subnational change assessments and monitoring in the context of national and international policy frameworks.

While climate and socio-economic factors are the most crucial variables for global livestock mapping, their influence varies significantly and often has complex relationships with modeled densities across species (Fig. 7). In contrast, our use of the potential land for livestock production layer—which integrates land use and land cover dynamics *via* high-resolution, Landsat-based data on cultivated/natural grasslands (Parente et al., 2024b) and croplands (Potapov et al., 2022b)—represents a fundamental methodological difference from the FAO GLW and AGLW products, which employ a more general and coarser suitability mask to determine where livestock can occur. Consequently, the major spatial differences between our output and the FAO GLW/AGLW data sets are primarily due to these constraints related to allocation of livestock species across the landscape.

### Overall applicability of spatiotemporal ML on census data modeling

We used a large reference sample set, composed of 55,336 census polygons and 939,257 individual data entries (in space and time), and 128 initial features for modeling. Despite being computationally intensive, our ML framework is fully automated and highly flexible. This automation allows us to generate updated global livestock layers within 24 h whenever new reference data from national/international agencies or new input raster layers become available (enabling predictions for 2023 onward). Despite that, mapping species densities and headcount at such a detailed resolution remains challenging in terms of computational effort, time to gather and harmonize input data, and conceptual work in designing a suitable modeling framework for complex livestock systems. Robust validation of such global prediction models is not trivial, as the differences in census data acquisition and quality across geographic regions is large. Even though our harmonized database covers 147 countries, several parts of Africa, Asia, Middle East and Oceania had limited census data, with temporal coverage of a few years (*i.e*., 2 to 4 years), unknown quality and/or only administrative units at level 2 available (Fig. 4). These inherent differences in quality, census polygon size and spatiotemporal coverage constitute a major limitation in producing further global livestock products at a finer resolution (*e.g*., 250 m resolution).

Furthermore, the mismatch between our modeling (at polygon level) and prediction scales (at 1 km raster cells) introduced a few shortcomings to the implemented framework. Once the algorithms (*i.e*., RF and GBT) and modeling strategies (*i.e*., BoxCox transformation and weighed samples) evaluated by us are non-linear, predicted values at 1 km might differ significantly from the mean expected densities at the polygon level. To address this issue, we adjusted the predicted values to match with the FAOSTAT national statistics, but this step can be executed using any other national/subnational census data as reference (as explained in subsection Usage Notes). Regarding the features, we noticed that when derived from spatially asymmetric/clustered layers they might have lower importance at modeling stage and significant impact in the output predictions. For instance, the human footprint layer (Mu et al., 2022) is not among the top-25 most important features in any of our livestock models (Fig. 6), however its spatial patterns (*i.e*., higher densities around road networks and distance to nearest cities) are visible in the output layers across different regions of the World (Figs. 9A–9C). This feature seems to have a stronger influence in dryland regions, where the homogeneity of important climate and terrain features results in lower predictive power from those variables. As an additional experiment, we removed the human footprint layer from the modeling and the CCC dropped by 8%, specifically for cattle densities.

Taking into account all these challenges, we decided to make our data and framework publicly available under the CC-BY license and then continue gradually improving predictions as more subnational data sets can be incorporated. We have made publicly available all input census data (raw and harmonized collations—https://doi.org/10.5281/zenodo.14926056) and output layers (https://doi.org/10.5281/zenodo.14933636) produced by this study. Our goal is to improve the usability, transparency and reproducibility of global livestock products, empowering various user and research communities to apply them in regional and local contexts (*e.g*., adjusting the headcounts based on data from national agencies; using national/regional mapping products of agriculture to refine the livestock spatial domain; estimating the upper and lower headcounts using prediction intervals; and modeling the density of livestock at the country level). In line with these possibilities, the following sections explore usage notes, potential applications, and the known limitations of our outputs.

### Usage notes

Our data open several possibilities to support environmental and agricultural applications, including, but not limited to: refining current estimates of greenhouse gas (GHG) emissions and nutrient cycles (De Azevedo et al., 2018), informing national livestock and land use policies (Mehrabi et al., 2020), contributing previously lacking time series inputs to global land use models (Venier-Cambron et al., 2024), estimating soil carbon sequestration rates (Dondini et al., 2023), as well as broader impacts on soil health (Byrnes et al., 2018). For applications relying on FAOSTAT headcount data, our global layers of annual headcounts serve as a fully compatible alternative, as the total sum of all mapped raster cells for a given country and year precisely matches the national estimates provided by FAOSTAT. On the other hand, this assumes that the FAOSTAT data are accurate, which is not always the case.

For subnational applications, we recommend obtaining headcount estimates based on the primary data provided by national census agencies. For example, our harmonized census database provides annual headcounts at the municipality level for Brazil (Brazilian Institute of Geography and Statistics, 2025) and the United States (U.S. Department of Agriculture, 2025) from 2000 to 2022 (*i.e*., full temporal coverage) and can be used to convert livestock density predictions into national calibrated annual headcounts. For countries without full temporal coverage (*e.g*., Canada, Argentina, India, Australia), the calibration can be based on a static adjustment factor, derived using all available years for each administrative division (Eq. (6)). Calibration can potentially run for multiple levels of administrative divisions (*i.e*., at the municipality, county, or country level) using more detailed national products for active grazing/forage areas (when available), resulting in a spatial explicit allocation of livestock species (1 km resolution) fully compatible with census data provided by national statistical agencies. For applications requiring more frequent temporal resolution (*e.g*., quarterly, monthly), our headcount estimates can be combined with intra-annual data sets of vegetation indices, precipitation and droughts, potentially supporting livestock practices in extensive systems (*e.g*., pastoralists in Sahelian West Africa) where seasonal cattle migration/transhumance is a key aspect (Ndiritu & Gichuki, 2025).

Based on 95% probability quantiles, our prediction intervals are relatively wide; therefore, for a more effective use, we recommend converting them to standard deviation (Eq. (7)) and deriving a prediction error around the predicted mean. To demonstrate this, the time-series of cattle predictions for the state of Rondônia in Brazil is shown in Fig. 10, including census polygons, headcounts, densities, prediction intervals, and prediction errors. At location lon = -64.0143 lat = -8.9694, we predict a total cattle density of around 174 heads per km^2^, with lower and upper prediction intervals of 11 and 450. Although the range is somewhat wide, if divided by 4, this gives a standard error of $\pm$100.

(7) $$SD \approx {{97.5th - 2.5th} \over 4}$$

where:

${{\mathrm{2}}}{{\mathrm{.5th}}}$ = Lower boundary, 2.5th quantile

${{\mathrm{97}}}{{\mathrm{.5th}}}$ = Upper boundary, 97.5th quantile

To evaluate this procedure, we estimated prediction envelopes for our testing set and the Prediction Interval Coverage Probability (PICP) (Tian et al., 2025) for all livestock species (showing PICP = 0.44/0.43/0.45/0.45/0.30 for cattle/horses/sheep/goat/buffalo). Additionally, it is important to note that the uncertainty might not be symmetrically distributed around the mean, since the livestock density follows the Poisson distribution, which is very skewed and with the majority of values concentrated around zero.

### Known limitations of output data

Our livestock modeling framework is highly dependent on the three key inputs: (i) livestock census data, (ii) potential land to allocate livestock production (modeling spatial domain), and (iii) detailed layers representing environmental and socio-economic drivers; without improving the quality of all three inputs, it is not possible to expect significant changes in the quality of the outputs. Although our predictions present global livestock estimates on very detailed scales (*i.e*., 1 km resolution and on annual basis), users should be aware of several limitations when using these data for decision-making or further applications. Currently, to our knowledge, the following limitations are among the most significant:

- **Livestock census data are often irregular, incomplete and overestimated**. Although advances in web technology have improved data accessibility, there are almost no standardized methods for recording, documenting, or distributing these data. Many historical records, especially before the 2010s, exist only in article form or PDFs, requiring careful interpretation, transcription, and translation. Within existing census data, some of them can be overestimated due to double counting. The sources of double counting arise from the mobility of livestock species (*e.g*., nomadic herds crossing census boundaries), outdated administrative records following changes in farm ownership or temporary animal placement, causing the same animal to be reported multiple times.
- **The availability of livestock census data varies widely per region**. In developed countries, administrative units are typically smaller, and most of the data is publicly accessible and easy to integrate through the Application Programming Interface (API). However, in more than half of the world’s landmass, our livestock census data is aggregated on large administrative scales (*e.g*., level 2), making it difficult to accurately allocate potentially very mobile species in our modeling framework.
- **Livestock headcounts are treated as ground truth**, which may not always be the case. We expect that, especially in developing countries, there might still be a lot of undocumented livestock so that, in general, our predictions might suffer from systematic underestimation of livestock counts. Currently, we are unable to determine the extent of this problem, as our livestock density models are trained and validated using census data, and we lack access to regions with a complete inventory of livestock species.
- **Livestock densities are based on two very different input data sets**—headcount estimates (census data) and active grazing/forage areas (EO data); resulting in unrealistic density values in specific cases. In countries where livestock production is dominated by feedlots and landless systems (*e.g*., the United States, West Europe), a high number of livestock species might not match to our layer of potential land for livestock production, resulting in very high densities (*e.g*., more than 2,500 cattle heads per km^2^). Additionally, the cropland mask (Potapov et al., 2022b) used in our modeling does not distinguish between food, feed, fiber, and other industrial crops, and has limited accuracy at national and regional levels (Kerner et al., 2024).
- **All potential land for livestock is used equally as a source of animal food for all livestock species**, which may not reflect the reality in some regions. A key limitation is that, despite the census polygon being the smallest unit with headcount information, livestock may not occupy all areas in the modeling spatial domain across all years. Our current model does not account for the complexities of different livestock management systems and species movement. For regions specifically dominated by landless systems, densities might not be determined by the variables we explored. Conversely, the model may overestimate densities in grazing lands within administrative units that contain high concentrations of confined, high-density farm animals. Furthermore, our layer of potential land for livestock production is used uniformly as input for all species. This issue probably impacts the global distribution of goats more severely, given that this species is primarily a browser.
- **Relatively standard machine learning models** might not be able to represent or incorporate complex relationships between environmental, socio-economic factors, and distribution of livestock species. We noticed that our models have problems in predicting areas with high values of livestock density, and eventually we are most likely underestimating hotspots of intense livestock density. Additionally, our prediction intervals have lower PICP, covering only between 30–40% of testing set, which means that our predictions intervals are too optimistic and should be used with caution.

### Future pathways for livestock mapping

**Figure 11:**
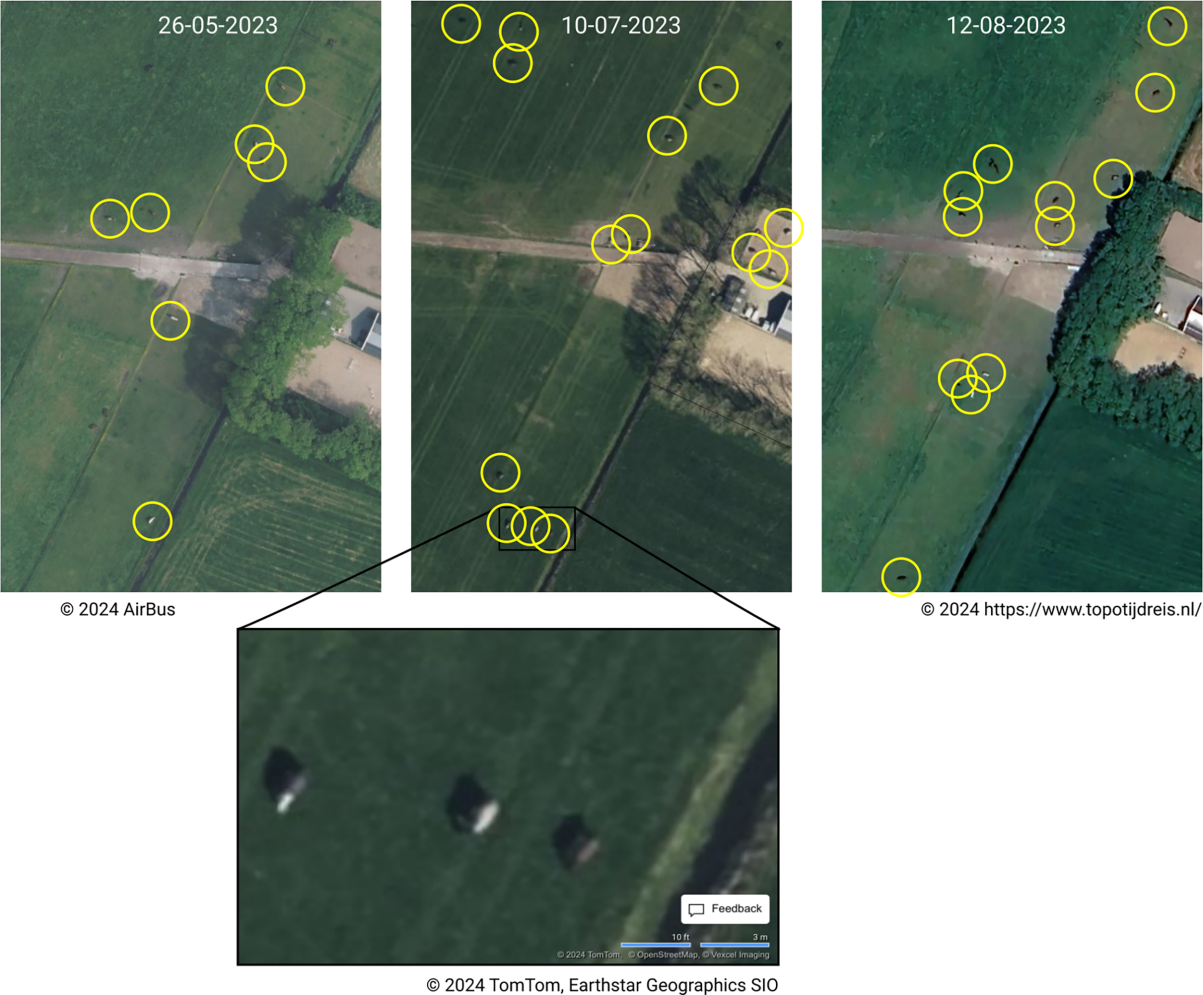
An example of cattle counting using very high-resolution images based on Google Maps, Microsoft Bing and Topotijdrijs.nl for a small area in the Netherlands (around Kampereiland/Municipality Kamper).

From a spatial statistics point of view, livestock species can be considered point-process-type variables (Pebesma & Bivand, 2023). Theoretically, it would be possible to count and digitize the locations of all species, even tracking their movements over an extended period, to create a complete inventory for a specific area. Hodel et al. (2026) show results of digitizing headcounts from Very High Resolution (VHR) satellite imagery (20–30 cm spatial resolution) for locations in Brazil. Even though recognizing livestock species in (VHR) imagery (*e.g*., Google Maps, Microsoft Bing, ESRI’s Wayback) is today possible (as also illustrated in Fig. 11), digitizing all livestock species worldwide would likely be impractical due to several factors: (i) VHR imagery may not be sufficient to distinguish between different livestock species (*e.g*., cattle and buffaloes; goats and sheep) (ii) livestock species are dynamic and migrate, so a single-day imagery snapshot may produce entirely inaccurate population estimates per farm or administrative area; (iii) in many countries, a substantial portion of livestock remains undetected in satellite imagery as species are kept in small enclosures, barns, or under tree cover (Malek et al., 2024).

Solutions of precision livestock farming (PLF) based on geolocation collars (Vidal-Cardos, Fàbrega & Dalmau, 2024) and face recognition system (Dac et al., 2022) might represent a more feasible way to produce a complete inventory of livestock species, although limited to farm level or small-scale regions. In addition, a better understanding of the spatial distribution of livestock production systems is essential to improve livestock distribution modeling. Herd parameters have the potential to help differentiate between dairy, cow-calf, and finishing systems, which are often regionally distinct. Furthermore, knowing the proportion of species housed indoors can refine distribution estimates by reducing over-representation (over-allocation) to grazing lands. Copernicus Sentinel-5P systems could potentially be used to address this problem, as they allow the production of independent emission products, such as the 2 km resolution gap-filled monthly values of nitrogen oxides (NOx) (Ho, Hengl & Parente, 2022) and methane emissions (Ho & Consoli, 2023), which could reveal hotspots of intense livestock density worldwide (Vanselow et al., 2024).

## Conclusion

Livestock plays a crucial role in global food systems, affecting interactions between society and nature while also influencing greenhouse gas emissions, biodiversity, and intensity of land use, which makes better livestock management essential for staying within the planetary boundaries (Herzon et al., 2024). Although our global predictions are limited in accuracy, we believe that the implemented modeling framework is a solid foundation for developing more accurate predictions in the near future. Because we have released all input/output data and source code under an open-data/open-source license, others can now refine and enhance these predictions to achieve higher accuracy at global, national, and subnational scales. One possible way to do this is to train the models per country and then integrate the results internationally. However, this was not possible in our case as we have more than 30% of countries with few or almost no data. In addition, some models for smaller countries could become unstable or have a low number of reference census data. Although it is justifiable that some countries prefer not to share detailed livestock statistics data, we believe that openly sharing these data benefits people across borders, multidisciplinary studies and cross-sector applications. We currently have widely accessible high-resolution data on human populations (Tiecke et al., 2017), while detailed information on livestock ownership remains limited, especially at the municipal level. Access to accurate data on deforestation, environmental degradation, greenhouse gas emissions, and livestock production is essential for informed decision making and the achievement of global sustainability goals (Pistora, 2024).

## Code and data availability

The data import and modeling presented in this article were implemented in R (LUCKINet—https://github.com/luckinet) and Python (scikit-map—https://github.com/openlandmap/scikit-map), and the source code is publicly available (MIT License) at: https://github.com/wri/global-pasture-watch. For reproducibility purposes and with the aim of contributing with various user and research communities, all livestock census data (raw and harmonized—https://doi.org/10.5281/zenodo.17665040; Parente et al. (2024a)), final ML models (https://doi.org/10.5281/zenodo.17665388; Parente et al. (2025a)), and output layers for potential land for livestock production (https://doi.org/10.5281/zenodo.14933679; Parente, Malek & Gonzalez Fischer (2025)), livestock densities, including prediction intervals and raw/uncalibrated headcount (cattle—https://doi.org/10.5281/zenodo.17486471; Parente et al. (2025c), goats—https://doi.org/10.5281/zenodo.17490112; Parente et al. (2025d), sheep—https://doi.org/10.5281/zenodo.17490692; Parente et al. (2025g), horses—https://doi.org/10.5281/zenodo.17490457; Parente et al. (2025e), and buffaloes—https://doi.org/10.5281/zenodo.17485929; Parente et al. (2025b)) and adjusted livestock headcount (FAOSTAT compatible—https://doi.org/10.5281/zenodo.17491242; Parente et al. (2025f)) are publicly available on Zenodo as open data (CC-BY license). Global livestock layers are also available in STAC (https://stac.openlandmap.org) and Google Earth Engine (https://developers.google.com/earth-engine/datasets/publisher/global-pasture-watch).

## Supplemental Information

10.7717/peerj.21494/supp-1 Supplemental Information 1 Livestock census data harmonized by Global Pasture Watch (GPW) in this study, organized by country, time coverage and administrative unit. Mean size of each administrative unit is presented together with the standard deviation. The start and end year refers mostly to national scale (level 1), thus most of sub-national entries have irregular temporal coverage.

## Additional Information and Declarations

### Data Availability

The following information was supplied regarding data availability:

The livestock census data is available at Zenodo: Parente, L., Ehrmann, S., Steffen, Cinardi, G., Wisser, D., Malek, Ž., Perez-Guzman, K., & Meyer, C. (2024). Global Pasture Watch—Livestock reference samples based on multi-source sub-national census data (2000–2024) (Version v1) [Data set]. Zenodo. https://doi.org/10.5281/zenodo.17665040.

The final ML models are available at Zenodo: Parente, L., Ehrmann, S., Bonannella, C., Malek, Ž., Gonzalez Fischer, C., Perez-Guzman, K., Stanimirova, R., Meyer, C., Wisser, D., Cinardi, G., & Sloat, L. (2025). Global Pasture Watch—Global machine learning models for livestock density predictions (Version v1). Zenodo. https://doi.org/10.5281/zenodo.17665388.

The potential land for livestock production maps are available at Zenodo: Parente, L., Malek, Ž., Ehrmann, S., Hengl, T., & Gonzalez Fischer, C. (2025). Global Pasture Watch—Annual maps of potential land for livestock production at 1-km for 2000–2022 (including production systems) (Version v1) [Data set]. Zenodo. https://doi.org/10.5281/zenodo.14933679.

The livestock densities are available at Zenodo:

- Cattle: Parente, L., Ehrmann, S., Hengl, T., Steffen, Bonannella, C., Malek, Ž., Gonzalez Fischer, C., Perez-Guzman, K., Stanimirova, R., Meyer, C., Wisser, D., Cinardi, G., & Sloat, L. (2025). Global Pasture Watch—Annual cattle density layers at 1-km for 2000–2022 (including 95% prediction interval) (Version v1) [Data set]. Zenodo. https://doi.org/10.5281/zenodo.17486471.

- Goats: Parente, L., Ehrmann, S., Hengl, T., Steffen, Bonannella, C., Malek, Ž., Gonzalez Fischer, C., Perez-Guzman, K., Stanimirova, R., Meyer, C., Wisser, D., Cinardi, G., & Sloat, L. (2025). Global Pasture Watch—Annual goat density layers at 1-km for 2000–2022 (including 95% prediction interval) (Version v1) [Data set]. Zenodo. https://doi.org/10.5281/zenodo.17490112.

- Sheep: Parente, L., Ehrmann, S., Hengl, T., Steffen, Bonannella, C., Malek, Ž., Gonzalez Fischer, C., Perez-Guzman, K., Stanimirova, R., Meyer, C., Wisser, D., Cinardi, G., & Sloat, L. (2025). Global Pasture Watch—Annual sheep density layers at 1-km for 2000–2022 (including 95% prediction interval) (Version v1) [Data set]. Zenodo. https://doi.org/10.5281/zenodo.17490692.

- Horses: Parente, L., Ehrmann, S., Hengl, T., Steffen, Bonannella, C., Malek, Ž., Gonzalez Fischer, C., Perez-Guzman, K., Stanimirova, R., Meyer, C., Wisser, D., Cinardi, G., & Sloat, L. (2025). Global Pasture Watch—Annual horse density layers at 1-km for 2000–2022 (including 95% prediction interval) (Version v1) [Data set]. Zenodo. https://doi.org/10.5281/zenodo.17490457.

- Buffaloes: Parente, L., Ehrmann, S., Hengl, T., Steffen, Bonannella, C., Malek, Ž., Gonzalez Fischer, C., Perez-Guzman, K., Stanimirova, R., Meyer, C., Wisser, D., Cinardi, G., & Sloat, L. (2025). Global Pasture Watch—Annual buffalo density layers at 1-km for 2000–2022 (including 95% prediction interval) (Version v1) [Data set]. Zenodo. https://doi.org/10.5281/zenodo.17485929.

The livestock FAOSTAT-adjusted headcount is available at Zenodo: Parente, L., Ehrmann, S., Hengl, T., Steffen, Bonannella, C., Malek, Ž., Gonzalez Fischer, C., Perez-Guzman, K., Stanimirova, R., Meyer, C., Wisser, D., Cinardi, G., & Sloat, L. (2025). Global Pasture Watch—Annual livestock headcount layers for cattle, goats, sheep, horses, and buffaloes at 1-km 2000–2022 (FAOSTAT-adjusted) (Part-1) (Version v1) [Data set]. Zenodo. https://doi.org/10.5281/zenodo.17491242.

All modeling framework is available at GitHub, Zenodo, STAC and Google Earth Engine:

- https://github.com/wri/global-pasture-watch.

- Parente, L., Ehrmann, S., & sarabandi, sajed. (2026). Global Pasture Watch—Source code of the global distribution of cattle, horses, goats, sheep and buffaloes at 1 km (Version v1). Zenodo. https://doi.org/10.5281/zenodo.20340463.

- https://stac.openlandmap.org.

- https://developers.google.com/earth-engine/datasets/publisher/global-pasture-watch.
