# Supplementary material for: Global distribution of cattle, horses, goats, sheep and buffaloes at 1 km resolution for 2000–2022 based on subnational census data and spatiotemporal machine learning
Source: PeerJ. 2026 Jul 17;14:e21494. doi: 10.7717/peerj.21494 (PMC13383963; doi:10.7717/peerj.21494)
Supplement: Supplemental Information 1 — Mean size of each administrative unit is presented together with the standard deviation. The start and end year refers mostly to national scale (level 1), thus most of sub-national entries have irregular temporal coverage. [file peerj-14-21494-s001.pdf]

Table S1. Livestock census data harmonized by Global Pasture Watch (GPW) in this study, organized by country, time coverage and administrative unit. Mean size of each administrative unit is presented together with the standard deviation. The start and end year refers mostly to national scale (level 1), thus most of sub-national entries have irregular temporal coverage.

#	Country	Time coverage					Sources	Administrative units		
		Cattle	Horse	Goat	Sheep	Buffaloes		Levels	Number of entries	Mean size
1	Afghanistan	2010 (1)	2010 (1)	2010 (1)	2010 (1)	NA	FAO	2	32	20095 ± 16708
2	Albania	2012—2020 (4)	2012—2018 (2)	2012—2020 (4)	2012—2020 (4)	NA	FAO, Malek et al., 2024	2,3	122	466 ± 364
3	Algeria	2001 (1)	2001 (1)	2001 (1)	2001 (1)	NA	FAO	2	48	48441 ± 115404
4	American Samoa	2003—2018 (3)	NA	NA	NA	NA	FAO	2,3	15	23 ± 34
5	Andorra	2011—2017 (7)	2011—2017 (7)	2011—2017 (7)	2011—2017 (7)	NA	FAO	2	7	67 ± 26
6	Angola	2005—2013 (9)	NA	2005—2013 (9)	2005—2013 (9)	NA	FAO	2	18	69720 ± 55300
7	Argentina	2008—2019 (12)	2008—2019 (12)	2008—2019 (12)	2008—2019 (12)	NA	GPW	3	549	5257 ± 6604
8	Armenia	2011—2019 (7)	2011—2019 (7)	2011—2019 (7)	2011—2019 (7)	2014 (1)	FAO	2	11	2699 ± 1433
9	Australia	2001—2020 (15)	2001—2011 (3)	2007—2016 (6)	2001—2020 (15)	2001—2011 (5)	FAO, GPW	3	701	22851 ± 101362
10	Austria	2000—2023 (23)	NA	2000—2023 (22)	2000—2023 (22)	2000—2023 (23)	GPW, Malek et al., 2024	3	1912	85 ± 758
11	Azerbaijan	2015—2019 (5)	2015—2019 (5)	2015—2019 (5)	2015—2019 (5)	2015—2019 (5)	FAO	2	80	2144 ± 3043
12	Belarus	2012—2017 (6)	2012—2017 (6)	NA	2012—2017 (6)	NA	FAO	2,3	124	3340 ± 7217
13	Belgium	2000—2023 (21)	NA	2000—2020 (10)	2000—2020 (11)	2000—2023 (21)	FAO, GPW, Malek et al., 2024	2,3,4+	602	253 ± 1052
14	Belize	2003—2015 (5)	2011 (1)	2003—2011 (2)	2003—2015 (5)	2011 (1)	FAO	2	6	3723 ± 1238
15	Benin	2000—2020 (15)	NA	2000—2020 (15)	2000—2020 (15)	NA	FAO	2,3	89	2614 ± 4776
16	Bhutan	2000—2018 (15)	2000—2018 (12)	2006—2018 (11)	2006—2018 (11)	2012—2018 (6)	FAO	2,3	220	343 ± 603
17	Bolivia	2001—2020 (20)	2008 (1)	2000—2013 (13)	2000—2013 (13)	NA	FAO	2,4+	336	6481 ± 26157
18	Botswana	2004—2015 (9)	2004—2015 (8)	2004—2015 (8)	2004—2015 (8)	NA	FAO, GPW	2,3,4+	55	31695 ± 40176
19	Brazil	2000—2022 (23)	2000—2022 (23)	2000—2022 (23)	2000—2022 (23)	2000—2022 (23)	GPW	3	6117	1585 ± 5747
20	Bulgaria	2011—2020 (10)	NA	2011—2020 (6)	2011—2020 (6)	2011—2015 (5)	FAO, Malek et al., 2024	3,4+	269	826 ± 2800

Continued on next page

Table S1 – continued from previous page

#	Country	Time coverage					Sources	Administrative units		
		Cattle	Horse	Goat	Sheep	Buffaloes		Levels	Number of entries	Mean size
21	Burkina Faso	2003—2019 (16)	2003—2019 (12)	2003—2019 (13)	2003—2019 (13)	NA	FAO, GPW	2,3	71	11615 ± 10508
22	Burundi	2005—2012 (7)	NA	2005—2012 (7)	2005—2012 (7)	NA	FAO	2	17	1595 ± 693
23	Cambodia	2003—2019 (6)	2013 (1)	2013—2017 (2)	2017 (1)	2009—2019 (3)	FAO	2	27	6762 ± 4706
24	Cameroon	NA	2017—2018 (2)	NA	NA	NA	FAO	2	10	46936 ± 30573
25	Canada	2011—2021 (3)	2011—2021 (3)	2011—2021 (3)	2011—2021 (3)	2011—2021 (3)	GPW	4+	2097	2505 ± 24034
26	Cape verd	2004 (1)	NA	2004 (1)	2004 (1)	NA	FAO	2	9	447 ± 300
27	Chad	2013 (1)	NA	2013 (1)	2013 (1)	NA	FAO	2	20	60934 ± 63726
28	Chile	2007—2021 (6)	2021 (1)	2007—2021 (5)	2007—2021 (5)	NA	GPW	2,3	58	21121 ± 28538
29	China	2000—2022 (23)	2000—2022 (23)	2000—2022 (23)	2000—2022 (23)	NA	GPW	2	31	303272 ± 373516
30	Colombia	2016—2024 (9)	2016—2024 (9)	2016—2024 (9)	2016—2024 (9)	2016—2024 (9)	GPW	3	2033	980 ± 3085
31	Congo	2014 (1)	NA	2014 (1)	2014 (1)	NA	FAO	2	12	28573 ± 20465
32	Costa Rica	2000—2014 (6)	2014 (1)	2014 (1)	2014 (1)	2014 (1)	FAO	2,3	86	1198 ± 2215
33	Croatia	2003—2020 (3)	2003 (1)	2003—2020 (3)	2003—2020 (3)	NA	FAO, Malek et al., 2024	2,3	573	197 ± 554
34	Cuba	2006—2016 (9)	2006—2016 (9)	2006—2016 (9)	2006—2016 (9)	2006—2011 (6)	FAO	2,3	135	1733 ± 2768
35	Cyprus	2000—2023 (23)	NA	2000—2019 (12)	2000—2019 (14)	2000—2023 (24)	GPW	2,3	2	9271 ± 9
36	Czechia	2000—2023 (22)	NA	2000—2020 (9)	2000—2020 (10)	2000—2023 (23)	GPW, Malek et al., 2024	3	22	7162 ± 4276
37	Denmark	2009—2023 (15)	NA	NA	2020 (1)	2009—2023 (15)	GPW, Malek et al., 2024	3	90	952 ± 2101
38	Ecuador	2000—2019 (6)	2000—2018 (5)	2000—2018 (5)	2000—2018 (5)	2012 (1)	FAO	2,3	222	2315 ± 4529
39	Egypt	2018 (1)	NA	2018 (1)	2018 (1)	2018 (1)	FAO	2	27	36487 ± 93871
40	El Salvador	2007 (1)	2007 (1)	2007 (1)	2007 (1)	NA	FAO	3	258	80 ± 86
41	England	2020 (1)	NA	2020 (1)	2020 (1)	NA	Malek et al., 2024	3	100	1279 ± 1477
42	Eritrea	2002—2010 (2)	NA	2002—2010 (2)	2002—2010 (2)	NA	FAO	2,3	62	3530 ± 6516
43	Estonia	2001—2020 (12)	2001—2018 (9)	2001—2020 (12)	2001—2020 (12)	NA	FAO, Malek et al., 2024	2,3	30	2955 ± 1014
44	Ethiopia	2003—2020 (14)	2003—2020 (14)	2003—2020 (14)	2003—2020 (14)	NA	FAO	2,3	209	18626 ± 35380

Continued on next page

Table S1 – continued from previous page

#	Country	Time coverage					Sources	Administrative units		
		Cattle	Horse	Goat	Sheep	Buffaloes		Levels	Number of entries	Mean size
45	Finland	2003—2023 (20)	2009—2011 (2)	2006—2020 (5)	2006—2020 (6)	2000—2023 (23)	FAO, GPW,Malek et al., 2024	3,4+	305	3443 ± 15036
46	France	2000—2023 (22)	NA	2001—2023 (20)	2001—2023 (20)	2000—2023 (22)	GPW	3	22	24943 ± 11400
47	French Guiana	2000—2010 (2)	NA	2000—2010 (2)	2000—2010 (2)	NA	FAO	3,4+	40	4138 ± 4606
48	Georgia	2006—2020 (15)	2014 (1)	2006—2020 (14)	2006—2020 (14)	2014 (1)	FAO	3,4+	76	1666 ± 2484
49	Germany	2000—2023 (11)	2016 (1)	2010—2016 (2)	2000—2023 (8)	2000—2023 (9)	FAO, GPW,Malek et al., 2024	3	473	2283 ± 4018
50	Ghana	2010 (1)	NA	2010 (1)	2010 (1)	NA	FAO	2	7	24428 ± 21727
51	Greece	2009—2020 (12)	2009—2019 (7)	2009—2020 (12)	2009—2020 (12)	2009—2020 (8)	FAO, Malek et al., 2024	2,3	146	2863 ± 3161
52	Guadeloupe	2000—2010 (2)	NA	2000—2010 (2)	2000—2010 (2)	NA	FAO	2	5	336 ± 384
53	Guam	2002 (1)	NA	2002 (1)	NA	2002 (1)	FAO	2	17	30 ± 26
54	Guatemala	2003—2007 (4)	2003 (1)	2003—2007 (2)	2003—2007 (2)	2003 (1)	FAO	2,3	351	620 ± 2257
55	Guinea	2001—2019 (9)	2001 (1)	2001 (1)	2001 (1)	NA	FAO, GPW	2,3	48	15381 ± 16127
56	Haiti	2009 (1)	NA	2009 (1)	2009 (1)	NA	FAO	2	10	2714 ± 1333
57	Hungary	2005—2020 (12)	2018—2020 (3)	2010—2020 (4)	2005—2020 (12)	NA	FAO, Malek et al., 2024	2,3	40	4650 ± 1820
58	Iceland	2011—2022 (9)	NA	2011—2021 (9)	2011—2022 (10)	2011—2022 (11)	GPW, Malek et al., 2024	3	8	25505 ± 31996
59	India	2003—2019 (4)	2003—2012 (3)	2003—2019 (4)	2003—2019 (4)	2003—2019 (4)	GPW	3	659	4765 ± 4475
60	Indonesia	2000—2022 (23)	2000—2022 (23)	2000—2022 (23)	2000—2023 (24)	2000—2023 (24)	FAO, GPW	2	62	60435 ± 72944
61	Iran	2005—2015 (3)	NA	2005—2015 (3)	2005—2015 (3)	2005—2015 (3)	FAO	2	59	55123 ± 58517
62	Iraq	2010—2018 (5)	2014—2018 (2)	2010—2018 (5)	2007—2018 (6)	2008—2018 (6)	FAO	2	18	24291 ± 29356
63	Ireland	2000—2023 (15)	2000—2010 (2)	2000—2020 (3)	2000—2023 (9)	2013—2023 (11)	FAO, GPW,Malek et al., 2024	2,3,4+	6235	55 ± 662
64	Italy	2000—2023 (21)	NA	2000—2023 (20)	2000—2023 (20)	2000—2023 (21)	GPW, Malek et al., 2024	3	7635	78 ± 846
65	Japan	2006—2017 (11)	2008—2013 (3)	2008—2013 (4)	2008—2013 (4)	NA	FAO	2	47	7964 ± 11038

Continued on next page

Table S1 – continued from previous page

#	Country	Time coverage					Sources	Administrative units		
		Cattle	Horse	Goat	Sheep	Buffaloes		Levels	Number of entries	Mean size
66	Jordan	2005—2017 (8)	2007—2017 (2)	2005—2017 (8)	2005—2017 (8)	NA	FAO	2,3	63	2767 ± 7026
67	Kazakhstan	2000—2013 (14)	NA	NA	2000—2013 (14)	NA	GPW	2	14	194905 ± 91913
68	Kenya	2009—2019 (7)	NA	2009—2019 (4)	2009—2019 (3)	NA	FAO	3,4+	469	3509 ± 7242
69	Kosovo	2020 (1)	NA	2020 (1)	2020 (1)	NA	Malek et al., 2024	3	33	288 ± 180
70	Kuwait	2001—2015 (15)	NA	2001—2015 (15)	2001—2015 (15)	NA	FAO	2	4	4324 ± 5680
71	Kyrgyzstan	2000—2013 (14)	2000—2012 (13)	NA	2000—2013 (14)	NA	FAO, GPW	2,3	54	11060 ± 12353
72	Laos	2005—2012 (4)	NA	NA	NA	2005—2012 (4)	FAO	2	18	12847 ± 4763
73	Latvia	2002—2020 (19)	2010 (1)	2010—2020 (2)	2002—2020 (19)	NA	FAO, Malek et al., 2024	2,3,4+	141	1365 ± 2416
74	Liechtenstein	2009 (1)	2009 (1)	2009 (1)	2009 (1)	NA	FAO	2,4+	12	24 ± 31
75	Lithuania	2005—2020 (8)	2005—2011 (7)	2005—2020 (8)	2005—2020 (8)	NA	FAO, Malek et al., 2024	2,3	70	1856 ± 2117
76	Luxemburg	2000—2023 (23)	NA	2000—2022 (9)	2000—2022 (11)	2000—2023 (23)	GPW, Malek et al., 2024	2,3	100	77 ± 361
77	Macedonia	2006—2020 (15)	2006—2020 (13)	2006—2020 (14)	2006—2020 (15)	NA	FAO, Malek et al., 2024	2,3	97	787 ± 1163
78	Malaysia	2011—2015 (4)	2007—2015 (2)	2011—2015 (4)	2011—2015 (4)	2011—2015 (4)	FAO	2	14	23702 ± 35040
79	Mali	2006—2014 (9)	2006—2014 (9)	2006—2014 (9)	2006—2014 (9)	NA	FAO, GPW	2	18	139807 ± 141830
80	Malta	2001—2023 (23)	NA	2001—2023 (17)	2001—2023 (18)	2001—2023 (23)	GPW	2,3	2	314 ± 0
81	Martinique	2000—2010 (2)	NA	2000—2010 (2)	2000—2010 (2)	NA	FAO	2,3	36	63 ± 87
82	Mexico	2004—2009 (6)	2007 (1)	2004—2009 (6)	2004—2009 (6)	NA	FAO	2	32	61475 ± 53733
83	Moldova	2016—2020 (5)	2018—2020 (3)	NA	NA	NA	FAO	3	35	822 ± 364
84	Mongolia	2002—2017 (15)	2002—2017 (15)	2003—2017 (14)	2003—2017 (14)	NA	FAO	2	22	71121 ± 45121
85	Montenegro	2010—2021 (11)	NA	2010—2021 (11)	2010—2021 (11)	2012—2022 (11)	FAO, GPW	2,3	22	1256 ± 2856
86	Morocco	2006—2022 (10)	2008 (1)	2006—2009 (4)	2006—2009 (4)	NA	FAO, GPW	2,3	64	15977 ± 21132
87	Mozambique	NA	2012 (1)	NA	NA	2011 (1)	FAO	2	12	65968 ± 38150

Continued on next page

Table S1 – continued from previous page

#	Country	Time coverage					Sources	Administrative units		
		Cattle	Horse	Goat	Sheep	Buffaloes		Levels	Number of entries	Mean size
88	Namibia	2008—2015 (7)	NA	2008—2015 (4)	2008—2015 (4)	NA	FAO	2	13	63718 ± 53074
89	Nepal	2011 (1)	2011 (1)	2011 (1)	2011 (1)	2011 (1)	FAO	2	5	29572 ± 8260
90	Netherlands	2020 (1)	NA	2020 (1)	2020 (1)	NA	Malek et al., 2024	3	418	98 ± 99
91	New Caledonia	2002 (1)	2002 (1)	2002 (1)	2002 (1)	NA	FAO	3	33	571 ± 323
92	New Zealand	2002—2022 (21)	2002—2022 (19)	2002—2022 (15)	2002—2022 (21)	NA	FAO, GPW	2,3	90	8454 ± 10120
93	Niger	2006—2017 (11)	2006—2017 (9)	2006—2017 (9)	2006—2017 (9)	NA	FAO, GPW	2,3	107	43567 ± 97837
94	Nigeria	2011 (1)	NA	NA	NA	NA	GPW	2	27	30871 ± 17546
95	Northern Ireland	2020 (1)	NA	NA	2020 (1)	NA	Malek et al., 2024	3	462	30 ± 43
96	Norway	2010—2020 (3)	2010—2012 (2)	2010—2020 (2)	2010—2020 (2)	NA	FAO, Malek et al., 2024	2,3	821	1346 ± 3210
97	Oman	2008—2010 (3)	NA	2008—2010 (3)	2008—2010 (3)	NA	FAO	2	8	38647 ± 41612
98	Pakistan	2006—2010 (2)	2006—2010 (2)	2006—2010 (2)	2006—2010 (2)	2006—2010 (2)	FAO	2,3	121	13175 ± 38923
99	Panama	2002—2011 (6)	2011 (1)	2011 (1)	2011 (1)	2011 (1)	FAO	2,3	77	1924 ± 2973
100	Paraguay	2008—2022 (5)	2008—2022 (4)	2008—2022 (4)	2008—2022 (4)	NA	FAO, GPW	2,3	234	3426 ± 10680
101	Peru	2000—2012 (12)	2012 (1)	2000—2012 (13)	2000—2012 (12)	NA	FAO	2,4+	1840	1412 ± 10485
102	Philippines	2011—2014 (4)	NA	2011—2014 (4)	NA	2011—2014 (4)	FAO	2,3	98	6313 ± 6558
103	Poland	2000—2023 (23)	NA	2000—2020 (11)	2000—2020 (12)	2000—2023 (23)	GPW, Malek et al., 2024	3	2684	343 ± 1555
104	Portugal	2000—2022 (23)	NA	2000—2022 (23)	2000—2022 (23)	2000—2022 (23)	GPW	3	7	13146 ± 13380
105	Puerto Rico	2002—2012 (3)	2002—2012 (3)	2002—2012 (3)	2002—2012 (3)	NA	FAO	2,3	86	210 ± 330
106	Qatar	2009—2014 (5)	2012 (1)	2009—2014 (5)	2008—2014 (5)	NA	FAO	2	9	1268 ± 1163
107	Reunion	2010 (1)	NA	2010 (1)	2010 (1)	NA	FAO	3	24	106 ± 63
108	Romania	2000—2020 (20)	2000—2018 (19)	2000—2020 (20)	2000—2020 (20)	2008—2012 (5)	FAO, Malek et al., 2024	2,3	83	5739 ± 1664
109	Russian	2007—2023 (17)	2007—2023 (17)	2007—2023 (17)	2007—2023 (17)	NA	GPW	3	1739	6751 ± 20474
110	Rwanda	2012 (1)	NA	2012 (1)	2012 (1)	NA	FAO	3	30	813 ± 424
111	Samoa	2009—2020 (2)	2009—2020 (2)	2009—2020 (2)	2009—2020 (2)	NA	FAO	2	1	2894 *pm*

Continued on next page

Table S1 – continued from previous page

#	Country	Time coverage					Sources	Administrative units		
		Cattle	Horse	Goat	Sheep	Buffaloes		Levels	Number of entries	Mean size
112	Saudi Arabia	2007—2015 (7)	NA	2007—2015 (7)	2007—2015 (7)	NA	FAO	2	13	148817 ± 152983
113	Senegal	2010—2012 (3)	2010—2011 (2)	2010—2012 (3)	2010—2012 (3)	NA	FAO	2	25	15834 ± 13882
114	Serbia	2012 (1)	2012 (1)	2012 (1)	2012 (1)	NA	FAO	2	25	3101 ± 973
115	Sierra Leone	2004—2015 (2)	NA	2015 (1)	2015 (1)	NA	FAO	2,3	17	8563 ± 8644
116	Slovakia	2004—2020 (9)	NA	2004—2011 (8)	2004—2020 (9)	2009—2010 (2)	FAO, Malek et al., 2024	2,3,4+	83	1771 ± 3252
117	Slovenia	2007—2023 (17)	NA	NA	2020 (1)	2007—2023 (17)	GPW, Malek et al., 2024	3	212	190 ± 1001
118	Somalia	2015 (1)	2015 (1)	2015 (1)	2015 (1)	NA	FAO	2	16	39836 ± 17901
119	South Africa	2005—2017 (9)	NA	2005—2017 (9)	2005—2017 (9)	NA	FAO	2	9	136083 ± 95622
120	South Korea	2012—2015 (3)	2012—2013 (2)	2013—2015 (2)	2013 (1)	NA	FAO	2,3	251	1185 ± 2967
121	Spain	2000—2023 (23)	NA	2000—2023 (22)	2000—2023 (22)	2000—2023 (23)	GPW, Malek et al., 2024	3	352	2877 ± 8951
122	Sudan	2007—2017 (10)	2011—2017 (7)	2008—2017 (9)	2007—2017 (10)	NA	FAO	2	28	88831 ± 84865
123	Sweden	2007—2023 (17)	NA	2008—2017 (4)	2007—2020 (8)	2007—2023 (17)	GPW, Malek et al., 2024	3	29	30893 ± 36164
124	Switzerland	2015—2023 (9)	NA	2020 (1)	2020 (1)	2015—2023 (9)	GPW, Malek et al., 2024	2,3	2062	59 ± 995
125	Syrian	NA	2008—2009 (2)	NA	NA	2008—2012 (5)	FAO	2	13	14500 ± 13989
126	Taiwan	2010—2019 (7)	NA	2010—2019 (7)	NA	2010—2019 (7)	FAO	2,3	42	2594 ± 5487
127	Tajikistan	2000—2014 (15)	NA	NA	2000—2014 (15)	NA	GPW	2	4	35539 ± 18681
128	Tanzania	2003—2017 (9)	2003—2008 (2)	2003—2017 (9)	2003—2017 (9)	NA	FAO	2,3	166	12990 ± 16773
129	Thailand	2013—2015 (3)	2009—2015 (6)	2007—2015 (9)	2007—2015 (9)	2007—2015 (9)	FAO	2	76	6816 ± 4774
130	Timor-leste	2013—2019 (3)	2013—2019 (3)	2013—2019 (3)	2013—2019 (3)	2013—2019 (3)	FAO	2,3	78	384 ± 418
131	Togo	2008—2016 (4)	NA	2008—2016 (5)	2008—2016 (4)	NA	FAO	2	5	11481 ± 4235
132	Tonga	2001—2015 (2)	2001—2015 (2)	2001—2015 (2)	2015 (1)	NA	FAO	2	5	135 ± 73
133	Tunisia	2002—2012 (5)	2004 (1)	2002—2012 (5)	2002—2012 (5)	NA	FAO	2	24	6483 ± 8185
134	Turkey	2009—2020 (8)	2011—2015 (5)	2009—2020 (8)	2009—2020 (8)	2010—2015 (6)	FAO, Malek et al., 2024	2,3	982	1590 ± 3122

Continued on next page

Table S1 – continued from previous page

#	Country	Time coverage					Sources	Administrative units		
		Cattle	Horse	Goat	Sheep	Buffaloes		Levels	Number of entries	Mean size
135	Turkmenistan	2000—2015 (16)	2001—2012 (6)	NA	2000—2015 (16)	NA	FAO, GPW	2	10	86434 ± 35510
136	Uganda	2000—2008 (2)	2008 (1)	2000—2008 (2)	2000—2008 (2)	NA	FAO	2	148	3159 ± 2473
137	Ukraine	2001—2019 (12)	2001—2019 (12)	2001—2017 (6)	2001—2019 (12)	NA	FAO	2	25	23999 ± 6519
138	United Arab Emirates	2012—2019 (7)	NA	2012—2019 (8)	2012—2019 (8)	NA	FAO	2	7	10215 ± 21934
139	U.K and Northern Ireland	2000—2021 (20)	2005—2021 (5)	2005—2021 (11)	2000—2021 (20)	2000—2021 (19)	FAO, GPW	2,3,4+	103	4881 ± 9188
140	United states	2000—2023 (24)	NA	2000—2017 (10)	2000—2023 (24)	2002—2017 (4)	GPW	3	6203	2748 ± 6239
141	Uruguay	2004—2017 (14)	2008—2012 (5)	2008—2014 (6)	2004—2017 (14)	NA	FAO	2	19	9403 ± 4006
142	Uzbekistan	2000—2014 (15)	NA	NA	2000—2014 (15)	NA	GPW	2	13	34429 ± 48568
143	Vietnam	2006—2014 (7)	NA	NA	NA	2006—2014 (7)	FAO	2	75	5282 ± 4195
144	Western Sahara	2009 (1)	NA	2009 (1)	2009 (1)	NA	FAO	2	2	134912 ± 54946
145	Yemen	NA	2007—2013 (7)	NA	NA	NA	FAO	4+	12	38010 ± 53734
146	Zambia	2016 (1)	NA	2016 (1)	2016 (1)	NA	FAO	2	9	83980 ± 44615
147	Zimbabwe	2001—2014 (12)	NA	2001—2014 (11)	2001—2014 (10)	NA	FAO	2,3	67	11702 ± 15440
